# Acquisition and establishment of the oral microbiota

**DOI:** 10.1111/prd.12366

**Published:** 2021-03-10

**Authors:** A. M. (Marije) Kaan, Dono Kahharova, Egija Zaura

**Affiliations:** ^1^ Department of Preventive Dentistry Academic Centre for Dentistry Amsterdam (ACTA) Vrije Universiteit Amsterdam and University of Amsterdam Amsterdam The Netherlands

**Keywords:** breastfeeding, delivery mode, infant, oral health, oral microbiota, plaque, puberty, saliva

## Abstract

Acquisition and establishment of the oral microbiota occur in a dynamic process over various stages and involve close and continuous interactions with the host and its environment. In the present review, we discuss the stages of this process in chronological order. We start with the prenatal period and address the following questions: ‘Is the fetus exposed to maternal microbiota during pregnancy?’ and ‘If so, what is the potential role of this exposure?’ We comment on recent reports of finding bacterial DNA in placenta during pregnancies, and provide current views on the potential functions of prenatal microbial encounters. Next, we discuss the physiological adaptations that take place in the newborn during the birth process and the effect of this phase of life on the acquisition of the oral microbiota. Is it really just exposure to maternal vaginal microbes that results in the difference between vaginally and Cesarian section‐born infants? Then, we review the postnatal phase, in which we focus on transmission of microbes, the intraoral niche specificity, the effects of the host behavior and environment, as well as the role of genetic background of the host on shaping the oral microbial ecosystem. We discuss the changes in oral microbiota during the transition from deciduous to permanent dentition and during puberty. We also address the finite knowledge on colonization of the oral cavity by microbes other than the bacterial component. Finally, we identify the main outstanding questions that limit our understanding of the acquisition and establishment of a healthy microbiome at an individual level.

## 1 INTRODUCTION

The first forms of life on Earth, about 2.9‐4 billion years ago, were anaerobic bacteria.^1^ Because Earth started as a bacterial planet, all eukaryotic forms of life, including the current plants, animals, and humans, have evolved in the presence of bacteria. The long history of shared ancestry and alliances between humans and microbes is reflected in their genomes. Analysis of the large number of full‐genome sequences presently available reveals that most life forms share approximately one‐third of their genes, including those encoding central metabolic pathways.^2^ Many human genes are homologues of bacterial genes that are mostly derived by descent, but occasionally by gene transfer, from bacteria.

**FIGURE 1:**
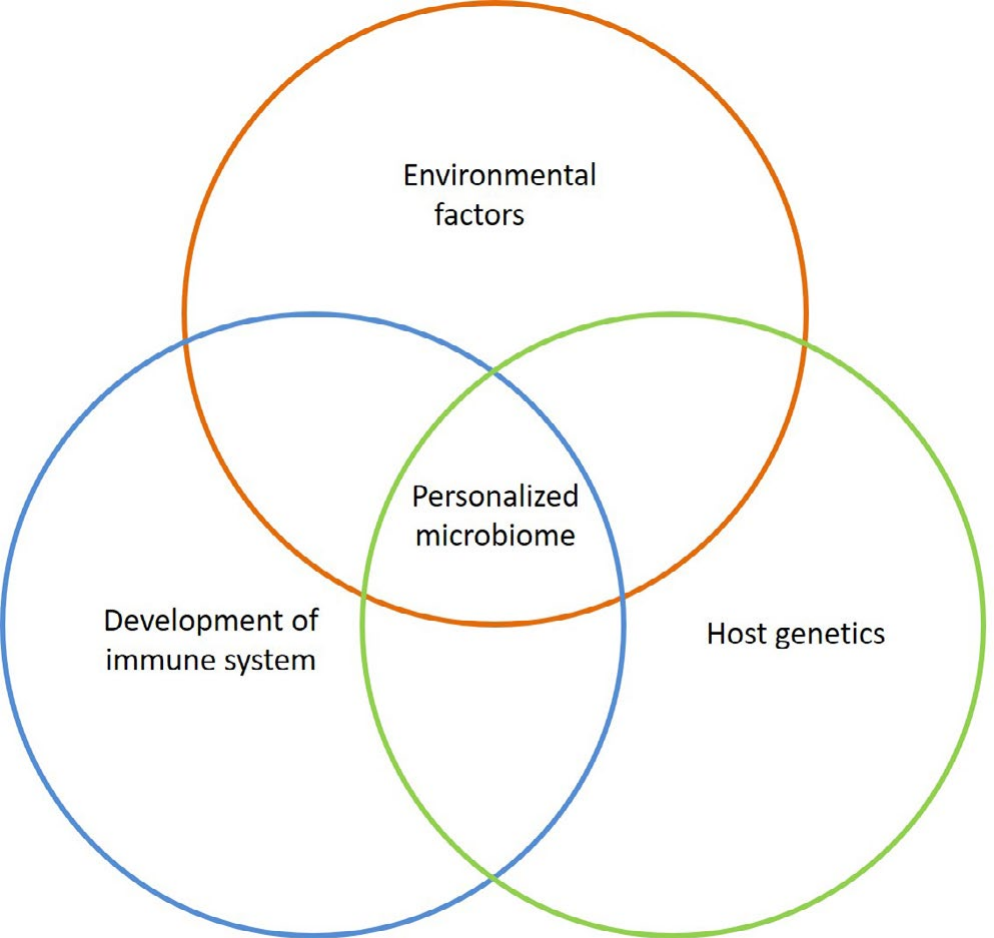
Interplay of factors contributing to acquisition and establishment of a personalized microbiome

Besides their common ancestry with microbes, humans have evolved in the continuous presence of, and in symbiosis with, microbes. The human body hosts approximately as many microbial cells as human cells,^3^ and the microbial cells on and in the body carry genes that outnumber human genes by at least a factor of 100.^4^ The human body lacks the specialized enzymes required for numerous chemical reactions (eg, nutrient breakdown) and therefore has to rely on its microbial symbionts to carry out these functions. As much as one‐third of the human metabolome (ie, the diversity of molecules circulating in blood) has a microbial origin.^2^ In return, microbes receive their favorite food from the host and a place to live. As a result of the mutualistic symbiosis there is continuous host‐microbiota crosstalk. Commensal microorganisms form the first line of defence and prevent exogenous microbes from becoming established: they train the immune system to recognize a “friend” from a “foe” by downregulating the pro‐inflammatory response toward commensals and upregulating this response against invaders.^5^ As a result of its indispensable functions for the host, the microbiome could be regarded as a well‐organized tissue of the body. Indeed, during health, at a functional level, the properties of the human microbiome are evenly distributed and prevalent across individuals and even across the various body sites.^6^ However, these global functions are performed by a highly personalized repertoire of microbiota, shaped by complex interplay between the genetic makeup and the immune system of the host under the influence of local and external environmental factors, such as exposure to microbes, the particular body niche, and the behavior of the individual host (Figure 1).

**FIGURE 2:**
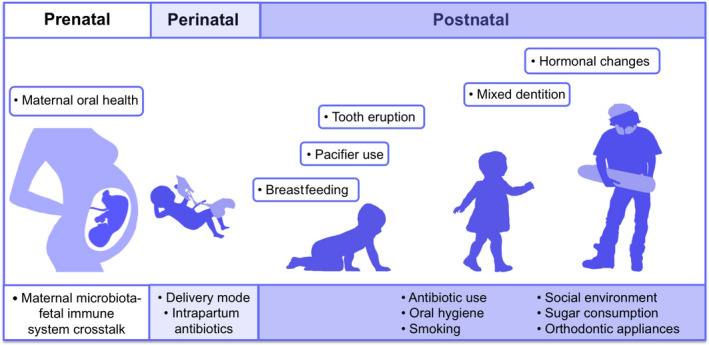
Factors shaping the oral microbiota from fetus through childhood and toward adolescence

Acquiring the microbial symbionts that would match the individual host is thus of utmost importance for the wellbeing of the individual. How does this process take place? How do humans pass on their microbes to their offspring? In this review, we address 3 chronological phases—prenatal, perinatal, and postnatal—from being a fetus, through childhood, and toward adolescence, and discuss the role of different host and environmental factors in acquiring and establishing the commensal oral microbiota (Figure 2).

## 2 PRENATAL PERIOD

The oral health and oral microbiota of a woman may directly affect her pregnancy and her developing fetus. A recent review of 23 systematic reviews on the relationship between maternal periodontitis and pregnancy complications concluded that if the mother has periodontal disease, she has a 1.6 (95% confidence interval: 1.3‐2.0) times higher risk for giving preterm birth, 1.7 (95% confidence interval: 1.3‐2.1) times higher ‐ for delivering a low‐birth‐weight infant, 2.2 (95% confidence interval: 1.4‐3.4) times higher ‐ for preeclampsia, and 3.4 (95% confidence interval: 1.3‐8.8) times higher ‐ for preterm birth plus delivery of an infant of low birth weight.^7^ It may seem more than reasonable to treat periodontal diseases during pregnancy in order to reduce these risks. However, currently there is insufficient evidence to conclude that periodontal treatment during pregnancy is effective in reducing the risks for adverse pregnancy outcomes.^8^ It has been proposed that periodontal pathogens or their by‐products reach the placenta and spread beyond it to the fetus.^9^

Recently the traditional dogma of sterile womb has been challenged by reports of microbiomes in placenta, amniotic fluid, umbilical cord blood, and meconium in complication‐free pregnancies.^10^, ^11^ Additionally, after analyzing over 300 placental biopsies and comparing the results with those of the Human Microbiome Project, for multiple body sites, Aagaard et al^12^ concluded that the placental microbiome resembles those of tongue and tonsils. The coevolution of the host and its microbes, and the knowledge that microbial symbionts in invertebrates are transmitted vertically,^13^, ^14^, ^15^, ^16^ makes one hypothesize about what the biological role of the placental microbiome might be.

It has been proposed that the placental microbiome is there to seed the fetus with microbes.^12^ However, assessment of the placental microbiome is performed by analysis of the bacterial DNA and not by isolation of bacteria. Finding culturable bacteria in meconium is used as an argument for microbial seeding in utero. A closer look at these studies shows that the presence of bacteria in meconium correlates with a longer time elapsed since delivery, thus indicating that bacteria are introduced at birth rather than during pregnancy.^11^ Delivery mode‐related differences in infant microbiota^17^ also oppose the hypothesis of intrauterine seeding. If microbiome was seeded in utero, it would not be possible to deliver axenic (germ‐free) mammals, including humans, by Cesarian section.^11^ Taken together, we can rule out the hypothesis that intrauterine microbial seeding is the biological function of the placental microbiome.

**FIGURE 3:**
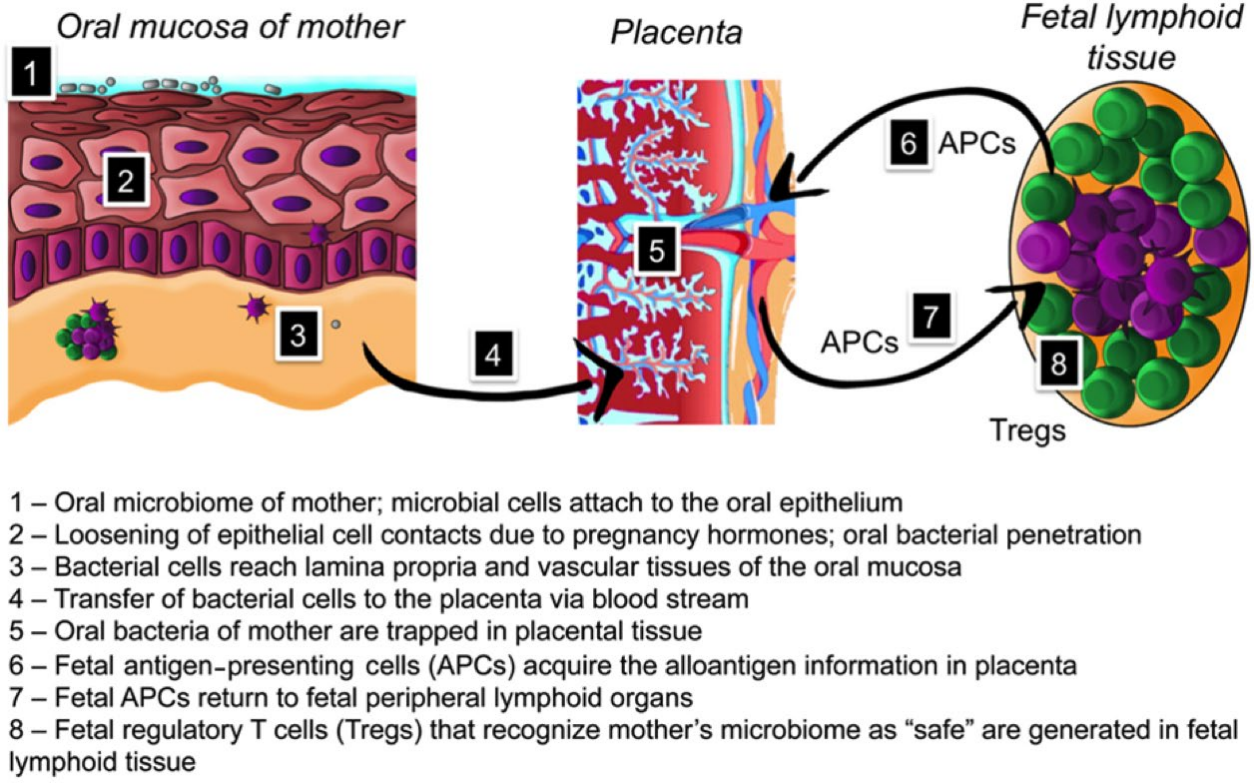
Hypothesis on the role of the placental microbiome in the development of fetal tolerance toward the (oral) microbiome of the mother, as proposed by Zaura et al^18^ (Figure with permission from the authors)

If bacteria or their fragments are transported to the placenta but are not seeding the fetus, what possible functions may they perform? Our group proposed that during pregnancy, the placenta becomes an antigen‐collecting site for the fetal immune system to be “trained” in antigen tolerance (Figure 3).^18^ Pregnant women develop increased gingival inflammation, also known as pregnancy gingivitis.^19^ This process is initiated by pregnancy hormones and leads to opening of the vascular bed, allowing hematogenic passage of oral microbes to placenta, either directly or by being engulfed and transported by the immune cells of the mother.^13^, ^15^ Microbial cells or their fragments are trapped in the placental tissue to be presented to the fetal immune system. In the prenatal period, fetal antigen‐presenting cells may interact with the mother's microbial antigens and return to fetal peripheral lymphoid organs. It has been shown that human fetal regulatory T cells become functionally suppressive after stimulation with maternal alloantigens and persist at least until early adulthood.^20^ If our hypothesis holds true, the fetus would develop prenatal tolerance to the microbiome of the mother and would regard it as “safe” during postnatal encounters with these bacteria. In other words, the development of fetal tolerance toward the microbiome of the mother during pregnancy could be the major factor for successful acquisition of a normal microbiome.^18^

Recently, Macpherson and his group demonstrated, through a series of elegant experiments with germ‐free mice, that the maternal microbiota indeed shapes the immune system of the fetus.^21^ To achieve gestation‐only colonization, they used a system in which pregnant germ‐free mice are transiently colonized with a genetically engineered strain of *Escherichia coli*. As this strain does not persist in the intestine, pregnant mice become germ‐free again before term and then deliver germ‐free pups. It was found that maternal colonization reprogrammed intestinal transcriptional profiles of the offspring, increased certain populations of innate lymphoid and mononuclear cells in the pups, and led to better avoidance of inflammatory responses to microbial molecules and penetration of intestinal microbes. Numerous radiolabeled (^13^C) bacteria‐derived molecules were passed from mother to offspring during pregnancy and these molecules (eg, natural microbial ligands for the aryl hydrocarbon receptor or their precursors) were present in maternal milk or in tissues of offspring. This study shows that many aspects of epithelial development and innate immunity, thus far thought to be part of normal development of newborns, are actually shaped through colonization by maternal microbes.^21^

To summarize the prenatal phase of microbiome acquisition: maternal microbiome is not passed directly to the fetus but prepares the fetus for postnatal microbial encounters by training and tuning the immune system of the developing fetus. Good maternal oral health and an oral microbiota in balance with the mother’s body are thus of utmost importance for a healthy child.

## 3 PERINATAL PERIOD

In utero, the fetus grows in a very safe environment with a constant temperature, is protected from microbial assaults, and receives nutrients and oxygen from the placenta via the umbilical cord. Following birth, all these factors change. At birth, the newborn immediately has to be able to regulate its thermal and respiratory homeostasis and glucose level, and it has to combat exposure to microbes. All these adaptations require modifications to the respiratory, metabolic, immune, and central nervous systems.^22^, ^23^

**FIGURE 4:**
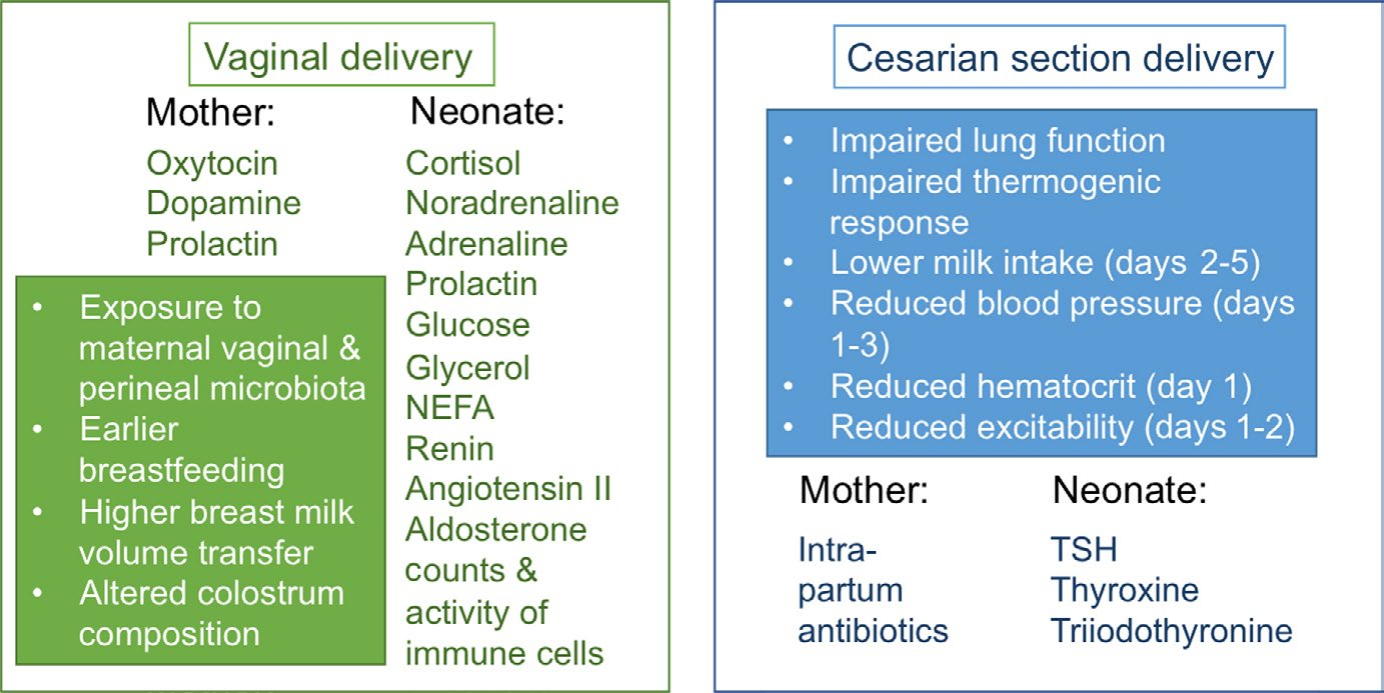
Summary of hormonal, metabolic, immunologic, and other physiologic differences during the perinatal period, stratified according to mode of delivery. Factors in green are higher during vaginal delivery and those in blue are higher after Cesarian section, as discussed by Hyde et al,^23^ either in the mother or in the neonate. NEFA, nonesterified fatty acids; TSH, thyroid‐stimulating hormone

Most infants are born by vaginal passage or vaginal delivery in the labor process. Some are delivered by Cesarian section, a life‐saving surgical operation that reduces the mortality of mother and/or child during delivery in medically compromised cases. In vaginal delivery, during passage through the vaginal canal, the fetus is compressed and the umbilical cord is occluded, leading to an increase in the level of stress hormones, of up to 20‐ to 100‐fold, in the neonatal blood.^24^ Cortisol and catecholamines are the primary mediators that prepare the fetus for birth and support the required physiologic adaptations.^22^ Besides increased levels of stress hormones, neonates delivered vaginally have higher levels of hormones involved in metabolism, blood pressure, and thermoregulation, leading to higher lipolysis, blood pressure, hematocrit, temperature, and appetite in the first few days after delivery than neonates delivered by prelabor Cesarian section (Figure 4).^23^ The immune phenotype is also affected: cord blood of vaginally delivered infants has higher counts and activities of immune cells and higher concentrations of a number of cytokines.^23^

Interestingly, the main substrate for a neonate’s microbiota—the breast milk of the mother—is also influenced by the delivery mode. The colostrum (breast milk collected within the first few days post‐delivery) produced after vaginal delivery has significantly higher antioxidative capacity than that produced after Cesarian section, in which an increased level of oxygen free radicals is observed, especially if the delivery is performed under general anesthesia.^25^ Additionally, total protein content in colostrum is significantly higher after vaginal delivery than after Cesarian section.^26^ Besides the chemical content, the microbiota of the colostrum is affected by the delivery mode.^27^, ^28^ Next to these compositional differences, the intake of breast milk by infants delivered by Cesarian section is lower, over the first 6 days of life, than by those delivered vaginally.^29^

At birth, vaginal delivery leads to direct exposure of the newborn to maternal vaginal and rectal microbes, while delivery by Cesarian section allows their microbes of the maternal skin and hospital environment to be the first to seed the neonate. Infants of <5 minutes of age were shown to have undifferentiated microbial communities on their skin and in their oral and nasal cavities, reflecting the delivery mode: vaginal microbiota, such as *Lactobacillus* species, *Prevotella* species, and *Sneathia* species, were dominant in vaginally delivered infants (*n* = 4), whereas skin‐associated microbes (*Staphylococcus* species, *Corynebacterium* species, *Propionibacterum* species) predominated after Cesarian section (*n* = 6).^17^ Within 2 days of delivery, saliva of Cesarian section‐ delivered infants (*n* = 53) had significantly lower bacterial counts and a lower prevalence of selected oral taxa (eg, *Actinomyces odontolyticus*,*Rothia dentocariosa*,*Bifidobacterium dentium*,*Streptococcus sanguinis*) than the saliva of those delivered vaginally (*n* = 95).^30^ This is in line with numerous studies on the microbiome of the infant gut, in which lower diversity and delayed colonization by certain microbiota is observed following a Cesarian section delivery.^31^

In a proof‐of‐principle study, 4 Cesarian section‐delivered neonates were exposed to maternal vaginal microbiota by rubbing their mouth, face, and the rest of their body with gauze incubated for 1 h in the vagina of their mother.^32^ The authors found that the microbiomes of Cesarian section‐delivered infants exposed to vaginal fluids resembled those of vaginally delivered infants, particularly during the first week of life.

Although delayed exposure to the vaginal and perianal microbiota of the mother during Cesarian section, as opposed to immediate exposure during vaginal delivery, is claimed to be the main reason for the differences observed in the microbiota, there is evidence that the microbiome of the infant is shaped not solely by the mode of delivery but also by antibiotics: buccal microbiomes of 3‐day‐old infants (*n* = 36) clustered not according to the mode of delivery but to maternal exposure to antibiotics during delivery.^33^ The oral microbiome of infants exposed intrapartum to antibiotic(s) had lower similarity to the maternal oral microbiome compared with unexposed neonates and contained higher proportions of several, mainly nonoral, taxa from the phylum Proteobacteria (*Bradyrhizobiaceae, Sphingomonadaceae, Comamonadaceae, Oxalobacteriaceae,* and *Neisseriaceae*); by contrast, bacterial families from the phylum Firmicutes (*Streptococcaceae,* and *Gemellaceae*) and the order *Lactobacillales* predominated in the unexposed neonates.^33^ As antibiotics are advised to be routinely used in Cesarian section deliveries,^34^ this might confound the microbial findings associated with the delivery mode.

A recent study from China described the effects of maternal vulval disinfection with povidone iodide—a common procedure performed during vaginal examination preceding vaginal delivery in that country—on the oral microbiome of newborns.^35^ Oral samples obtained from 10 infants immediately after Cesarian section and from 20 infants born by vaginal delivery (of which 10 were preceded by povidone iodide disinfection of the vulva) were compared. Infants delivered vaginally with no vulval disinfection step had the lowest oral bacterial diversity and their oral microbiome was dominated by bacteria of the genus *Lactobacillus*, while both Cesarian section‐delivered infants and those delivered vaginally after vulval disinfection lacked this genus and harbored a significantly more diverse microbiome with higher proportions of several genera, including *Prevotella*, *Escherichia*,*Shigella*, *Staphylococcus*, and *Klebsiella*.

Based on the available studies, there is consensus that the delivery mode‐related differences in the oral microbiota are clearly evident in the first 3‐8 months of life,^30^, ^36^, ^37^ and at the individual taxa level they are still discernable in children at least up to 4‐5 years of age,^38^, ^39^ and perhaps even longer. Saliva of infants delivered vaginally (*n* = 73), followed from birth through 1, 3, and 6 months of age, demonstrated greater diversity at all sampling time points, and showed closer similarity to the salivary microbiota of the mother than saliva of infants delivered by Cesarian section (*n* = 44).^30^ Higher diversity of salivary microbiota in the vaginal delivery group was confirmed in a study on saliva of 3‐month‐old infants.^36^ *Slackia exigua* was exclusively found in the infants delivered by Cesarian section (*n* = 38); these infants also showed a higher prevalence of certain lactobacilli and *Streptococcus parasanguinis*, and a lower prevalence of *Haemophilus parainfluenzae,* *Leptotrichia/Sneathia*, *S. sanguinis*, and *Cardiobacterium* than vaginally delivered infants (*n* = 25).^36^ Another study, of a cohort of 83 Irish infants (43 of whom were delivered by Cesarian section), confirmed significant differences in diversity and microbial composition of saliva in the first week since delivery, while in the follow‐up samples, collected at weeks 4 and 8 and at months 6 and 12, the difference according to delivery mode was lost.^40^ A recent study on the salivary microbiome of Swedish children, followed from birth until the age of 7, did not confirm the differences in microbial diversity, but did find significant delivery mode‐related differences at the microbial profile level up to the age of 6 months, followed by a convergence in similarity of the microbial profiles over time.^41^ At the age of 7 years, quite in contrast to the direction of the finding from the study, described above, on 3‐month‐old infants,^36^ a significantly higher proportion of genus *Haemophilus* was found in saliva of children delivered by Cesarian section (*n* = 12) compared with children delivered vaginally (*n* = 68).^41^ This controversy again indicates that the sample size of these studies ^36^^,^^41^) might have been too low to assess this issue reliably. Another plausible explanation could be the different microbial detection methods used in the 2 studies.

The most commonly studied oral microorganism in infants and children is certainly *Streptococcus mutans*. Intriguingly, there is no consensus on the relationship between the prevalence of this caries‐associated microorganism and the delivery mode: some studies did not find any relationship,^37^, ^42^ whereas others found a higher prevalence of *S. mutans* in the vaginally delivered group.^30^, ^39^ The findings of the only longitudinal study on this topic contradict the results of those studies described above: in this study, mother‐infant pairs (127 underwent vaginal delivery and 29 underwent Cesarian section delivery) were followed from birth until the children were 4 years of age; children in the Cesarian section group acquired *S. mutans* at a younger age (17.1 months) than children in the vaginal delivery group (28.8 months).^38^ Larger longitudinal studies are necessary to dissect this issue.

Cesarian section is a life‐saving operation. However, at a population level, the association between Cesarian section and the decrease in mortality outcomes is lost if the rate of Cesarian section is above 9%‐16%.^43^ In some countries, the Cesarian section rates are reaching epidemic proportions. In 2015, Cesarian section was performed in 21.2% of live births globally, in 44.3% of all deliveries in Latin American and Caribbean regions, with Brazil hitting the top with a Cesarian section rate of 56%.^44^ These extremely high Cesarian section rates are alarming because epidemiological studies show that children delivered by Cesarian section have higher risk for immunological disorders and diseases, such as asthma, allergic rhinitis, wheezing, allergic sensitization, food allergy, systemic connective tissue disorders, juvenile arthritis, inflammatory bowel diseases, immune deficiencies, leukemia, obesity and type 1 diabetes, compared with children delivered vaginally.^45^ Only a few studies have looked into the potential mechanisms behind these epidemiological findings. For instance, Cesarian section‐delivered Finnish infants were shown to have a stronger nonspecific humoral immune response: they had higher total numbers of IgA‐, IgG‐, and IgM‐secreting cells in blood than their vaginally delivered counterparts throughout the first year of life.^46^ Taken together, the mode of delivery might influence the maturation of the immune system and affect the programming of long‐term health.

In summary, transition from prenatal to postnatal life involves multiple crucial adaptations in respiratory, metabolic, immune, and central nervous systems of the neonate. These adaptations, together with exposure to antibiotics and maternal microbes, are highly influenced by the mode of birth, which in turn is shown to affect the perinatal period and might even have long‐lasting effects on the general health of the individual.

## 4 POSTNATAL PERIOD

While the previous section focuses on the first days of neonatal life, here we address the factors contributing to the transmission and niche‐specificity of the oral microbiota, the role of dietary and behavioral habits, the effect of medication, current knowledge on the influence of the genetic background, changes from deciduous to permanent dentition, and hormonal effects during puberty, on the acquisition and establishment of the oral microbiota throughout childhood and toward adolescence.

### 4.1 Microbial trafficking between the oral cavity and other sites in the body

Shortly after birth, the neonatal microbiomes are homogenous among gut, oral, nasal, and skin communities.^47^, ^48^, ^49^ The oral microbiome in low‐birth‐weight newborns was shown to seed the gut microbiome, which diverged within 2 weeks to a gut‐specific community.^47^ Microbiota change over time, with body sites serving as a primary determinant of the composition of the microbial community and its functional capacity.^48^ This is probably driven by differences in the local environment. The oral microbiota in 6‐month‐old infants exhibit high similarity with the microbiota of their mothers’ oral cavity, breast milk, and mammary areola.^49^ The same tendency was shown between skin and gingival microbiota, with the microbiota of the nares as a bridge between the two.^48^ These results show that constant contact between microbial communities influences their composition.

As a result of its liquid nature, saliva is thought to serve as a carrier of microbiota and to transport microorganisms from one body site to another. In adults, most bacteria are inactivated in the acidic environment (pH 1‐2) of the lumen of the stomach. However, the gastric pH of infants is higher because of the combined effects of a high buffering capacity and the high pH of human milk: the average prefeeding gastric pH measured in 25 full‐term healthy 5‐ to 13‐day‐old infants was 3.5 (range: 2‐6.1), and it rose above pH 6 (range: 5.2‐7.1) within 30 minutes of starting a feed, thereafter remaining above pH 4 for 2‐4 hours.^50^ This probably leads to the influx and establishment of bacteria in the gut. In adult patients who use proton pump inhibiting medication for treating stomach ulcers, gastric pH is also increased. In these individuals, the abundance and diversity of gut commensals was reduced, with an associated significant increase in the abundance of oral commensals in the gut.^51^

Recently it was shown that hands function as an important vector for the transfer of oral and fecal microbes within families.^52^ The palms of the hands of infants (<2 years of age) have a higher proportion of tongue bacteria than the palms of the hands of children aged 2‐18 years, with the proportion of tongue bacteria decreasing with increasing age; by contrast, the proportion of palm bacteria sourced from stool increases with increasing age.

Medication use is shown to disturb the development of a normal microbial community and to promote the spread of oral taxa outside their normal habitat. Infants who experienced multiple respiratory tract infections in their first year of life and received antibiotics to treat these infections showed aberrant development of the nasopharyngeal microbial community compared with the control group.^53^ These aberrant microbial communities were less stable and were enriched with oral taxa, including *Neisseria* and *Prevotella*, while children with higher stability of their nasopharyngeal microbial communities were more resistant to respiratory tract infections.

### 4.2 Single oral cavity—multiple distinct microbial niches

**FIGURE 5:**
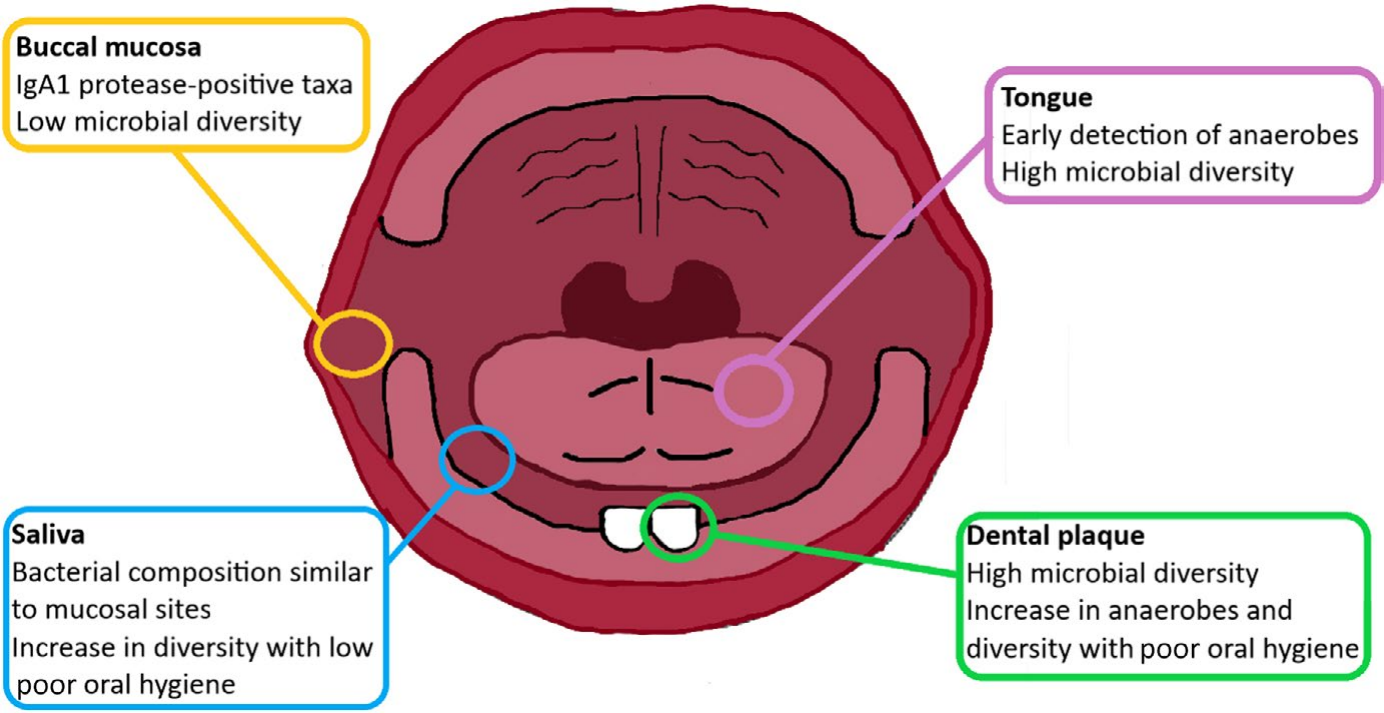
Niches in the infant oral cavity and their main microbial characteristics

The famous words of the Dutch microbiologist Baas‐Becking: “Everything is everywhere but the environment selects”^54^ apply to any ecosystem, including the oral cavity. The local environment (eg, the structure of the surface to which bacteria adhere, and the availability of oxygen and nutrients) influences the composition of the microbiota. This is reflected in finding distinct microbial communities at different oral niches, such as tongue, buccal mucosa, supragingival plaque, and subgingival plaque.^55^, ^56^, ^57^ The dorsum of the tongue, for example, with its papillae and crypts, provides optimal conditions for strict anaerobes. Similarly, eruption of the teeth can be seen as a milestone in the development of microbial communities because it creates a unique, nonshedding surface for accumulation of both supra‐ and subgingival plaque (Figure 5), and leads to an increase in microbial diversity.^55^

As a gateway to the gastrointestinal tract, the oral cavity harbors a unique environment, which can serve as a safe haven or a short‐term parking site for microorganisms. Whether microorganisms traverse the oral cavity without attaching to an oral surface depends on their ability to adhere. Adherence between a microorganism and a surface, or between 2 microorganisms, takes place when a receptor (usually a salivary or a bacterial component)^58^ and an adhesin (usually a cell‐wall component, fimbriae, or 2 adhesins) connect.^59^ The oral cavity contains 3 distinct types of surfaces for microorganisms to adhere to: the teeth; the oral mucosa; and other bacteria (through a process called coaggregation).

After the tooth surface is cleaned, it becomes covered with a thin proteinaceous layer, called the acquired enamel pellicle. This pellicle consists of proteins (eg, statherin, histatin, albumin, acidic proline‐rich‐proteins), enzymes (eg, amylase, lysozyme, peroxidase), glycoproteins and mucins (mucin‐1, mucin‐2, mucin‐5B), and lipids.^60^ Early colonizers, such as streptococci and actinomycetes, are able to adhere to specific molecules in this pellicle. By doing so, they become an attachment site for other microorganisms, such as *Fusobacteria, Veillonella,* and *Rothia*.^61^ Less well studied is the mucosal pellicle, the main components of which are mucins (mucin‐5B and mucin‐7) and secretory IgA.^60^ This mucin‐rich layer covers the mucosal surfaces of the oral cavity and its physiological role in, for example, maintaining mucosal integrity and promoting or preventing microbial colonization of mucosal surfaces, is still to be disclosed.

Besides promoting the adhesion of bacteria, there are several mechanisms that are aimed at preventing the colonization of microorganisms in the oral cavity. One such mechanism involves secretory IgA, an antibody delivered by salivary secretion and concentrated within the mucosal pellicle.^60^ Secretory IgA binds to and blocks bacterial adhesins, precluding adherence of bacteria to the surfaces.^62^ There are 2 subclasses of secretory IgA—IgA1 and IgA2—with saliva and human milk containing mainly IgA1.^63^ The hinge region of the IgA1 chain is protected from traditional proteolytic enzymes. However, a number of bacterial pathogens (eg, *Neisseria meningitidis*, *Streptococcus pneumoniae*) and some oral commensals (eg, *S. sanguinis*, *Streptococcus oralis, Prevotella* species, and *Capnocytophaga* species) produce highly specific IgA1 proteases that are able to cleave IgA1 in the hinge region. In vitro studies have shown that bacteria expressing these proteases are able to negate the inhibitory effect of secretory IgA on their adherence^63^; thus, they possess a colonization advantage. A longitudinal study on 50 infants followed from 2 to 24 months of age assessed their oral streptococci and found that all infants harbored IgA1 protease‐positive streptococci at 2 years of age and, moreover, that IgA1 protease activity was already present in salivary isolates from 76% of the infants when they were 2 months of age.^64^ The main species that produce IgA1 protease are *Streptococcus mitis* biovar 1 (especially during the first year of life), *S. oralis*, and *S. sanguinis*. By inhibiting certain species of bacteria and promoting the establishment of others, the immunological actions of the host influence bacterial colonization, exemplifying the symbiotic evolution of humans together with their microbes.

Saliva, in this sense, has quite an ambivalent function. On the one hand, it carries the microorganisms throughout the mouth to potential colonization surfaces and it contains adhesion‐promoting components. On the other hand, saliva contains IgA, which prevents microorganisms from adhering, and aids the removal of microorganisms during swallowing. In addition, salivary flow may mechanically affect the colonization of oral surfaces. A recent study in which over 9000 oral samples were analyzed shows that, regardless of tissue type (teeth, alveolar mucosa, keratinized gingiva, or buccal mucosa), surface‐associated bacteria vary along an ecological gradient from the front to the back of the mouth.^65^ These results imply that salivary flow influences the spatial organization of microbial communities.

Only a few studies have assessed the microbial composition of multiple distinct oral niches in infants (Table 1) and the majority demostrate limitations, such as small sample size, studying specific microorganisms only, or the absence of longitudinal data. Based on these limited findings it is clear that more than 1 oral niche should be sampled because sampling only saliva^66^ or plaque,^67^ for example, does not reveal all genotypes of *S. mutans* and *S. sobrinus*, and therefore is not representative of the entire oral ecosystem. A recent longitudinal study characterized maturation of the salivary and dental plaque microbiome in 119 caries‐free children and their primary caregivers^68^; it was demonstrated that, in 1‐year‐old children, the salivary microbiome had a significantly higher number of bacterial taxa than dental plaque. In the follow‐up samples, collected when children reached 2.5 and 4 years of age, the diversities of both niches were comparable but significantly higher than the diversity of samples collected from children when 1 year of age. Overlap in taxa (determined using zero‐radius operational taxonomic units) between the overall saliva and plaque datasets increased with age, from 72% in 1‐year‐old children to 83% in 4‐year‐old children. Interestingly, within an individual child, the shared proportion of zero‐radius operational taxonomic units between plaque and saliva varied greatly: in a single child there were no shared zero‐radius operational taxonomic units at all, while the maximum overlap between the individual paired saliva and plaque samples collected at the same time point was 65% zero‐radius operational taxonomic units.

**TABLE 1:** Studies assessing microbial composition of multiple oral niches in infants

Reference	Study design	Study population	Sample type	Microbial analysis	Author’s conclusions and reviewer’s comments
Grönroos et al^66^	Cross‐sectional	7 children (3‐7 y)	Supragingival plaque (from caries‐free enamel and caries lesions); saliva (5 children)	Culture, PCR	*S. mutans* colonizes hard tissues. Saliva sample does not necessarily reveal all genotypes. When assessing mutans streptococci, it is necessary to sample multiple oral sites Small sample size; only *S. mutans* assessed
Klein et al^67^	Longitudinal cohort (20 mo follow‐up)	16 mothers, 16 infants (mean age: 5.9 ± 1.5 mo)	Mothers: saliva; Children: saliva, tongue dorsum, alveolar ridge mucosa, supragingival plaque	Culture, PCR	The majority of *S. mutans* and *S. sobrinus* genotypes and strains were isolated from dental plaque. Dental plaque alone was not representative of all genotypes detected in the children’s oral cavity Only *S. mutans* and *S. sobrinus* assessed
Lindquist et al^69^	Longitudinal (birth to 7 y)	15 mothers and newborns	Mothers: saliva; Children: saliva, tongue dorsum, supragingival plaque	Culture, ribotyping	*S. mutans* and *S. sobrinus* were detected earlier in saliva than in dental plaque or on the tongue. There was a tendency for *S. sobrinus* to be detected earlier in saliva than in plaque or tongue. Saliva could be a source of microbes that seeds the other niches Only *S. mutans* and *S. sobrinus* assessed
Milgrom et al^70^	Cross‐sectional	179 children (*n* = 45, 6‐12 mo; *n* = 86, 13‐24 mo; *n* = 48, 25‐36 mo)	Supragingival plaque (from caries‐free enamel or caries lesions), tongue dorsum	DNA‐DNA hybridization	There was a tendency for earlier colonization of tongue than of supragingival plaque by*S. mutans* and *S. sobrinus*. *S. sobrinus* was found on tongue more often in the youngest age group, but in supragingival plaque more often in the oldest age group Only *S. mutans* and *S. sobrinus* assessed
Tanner et al^71^	Cross‐sectional	171 children (*n* = 57, 6‐18 mo; *n* = 114, 19‐36 mo)	Supragingival plaque (from caries‐free enamel or caries lesions), tongue dorsum	DNA‐DNA hybridization	Detection rates of *S. mutans* (70%), *S. sobrinus* (72%)*, P. gingivalis* (23%), *B. forsythus* (11%), and *A. actinomycetemcomitans* (30%) in tongue samples were similar in children 6‐18 and 19‐36 mo of age. Strong association was found between species detected in supragingival plaque and tongue . Most species were more frequently detected on tongue than on supragingival plaque in 6‐ to 18‐mo‐old children, suggesting that the tongue is a potential microbial reservoir

In addition, age‐related differences in the colonization of specific pathogens at oral sites have been observed. *Streptococcus mutans* and *S. sobrinus* were found earlier in saliva than on the tongue or in plaque of children^69^ and the presence of *S. sobrinus* on tongue surfaces decreased with increasing age of the child, whereas its presence in supragingival plaque increased over time.^70^ In a cross‐sectional study, most species were detected more frequently on the tongue than in dental plaque, suggesting that the tongue is a potential microbial reservoir.^71^

Samples of saliva are easy to obtain, and saliva is not a niche per se but rather a mixture of microorganisms dislodged from various oral niches. The total surface area of the oral cavity of 5‐year old children with a full primary dentition is estimated to be about 118 cm^2^; teeth represent 13% of the surface area and mucosal surfaces represent the remaining 87% (12% palate, 22% gingival and alveolar mucosa, 26% buccal mucosa, 13.5% ventral surface of the tongue and floor of the mouth, and 13.5% dorsum of the tongue).^72^ Even from these conservative estimates, in which the area for microbes to attach (eg, within the crypts of the dorsum of the tongue) has been underestimated, it is clear that dental surfaces are by far outnumbered by the mucosal area. The difference in area between mucosal surfaces and teeth is the most likely explanation of why salivary microbiota resembles those from mucosal surfaces and not those from teeth: data from the Human Microbiome Project, in which microbiomes of different intraoral niches in over 200 adults were compared, demonstrates that bacterial profiles of saliva are more similar to those of mucosal sites (tongue, tonsils, hard palate) than to those of dental surfaces (supragingival and subgingival plaque).^73^

In summary, the mouth is a complex ecosystem with multiple distinct niches and microbial habitats. The oral cavity of infants has not been as extensively sampled as that of adults, and large‐scale longitudinal studies on multiple oral niches are lacking. Although several methods have been used to collect oral samples from infants (eg, saliva, buccal mucosa, tongue or alveolar ridge swabs), it remains unclear which method or which combination of methods would give the most informative results.

### 4.3 Acquiring specific oral microbial taxa

As already mentioned, because of its association with caries, *S. mutans* is one of the most investigated oral microorganisms in infants. Its source and colonization have been the scope of numerous studies. Two ‘windows of infectivity’ for colonization with *S. mutans* have been proposed: the first between 19 and 31 months of age^74^; and the second after eruption of the first permanent molars.^75^ Colonization with *S. mutans* might occur earlier in individuals with high caries risk, as shown in a longitudinal study on American Indian children, in which 58% of the children had *S. mutans* by the age of 16 months.^76^ Although not common in modern society, in more traditional cultures the mother premasticates the food for her infant.^77^ A study on *S. mutans* colonization found that food prechewing, together with high maternal salivary *S. mutans* counts, were associated with increased colonization of infants with this microorganism.^39^

Although *S.mutans* preferably colonizes teeth, its DNA has been found in tongue samples from predentate infants^71^ and it has been repeatedly cultured from swabs of the alveolar ridges of infants before tooth eruption,^78^, ^79^ providing evidence that this microorganism can also colonize mucosal surfaces.

Infants acquire *S. mutans* primarily by vertical transmission from their mother, although horizontal transfer from other caretakers and family members and from children in nurseries also occurs.^80^, ^81^, ^82^ To identify strain‐level relatedness, different strain‐typing methods are available.^83^ Depending on the typing method used, mother‐child transmission of *S. mutans* has been shown to range from 50% to 85%, with multilocus sequence typing being the most discriminatory among the methods.^84^

Factors that promote transmission of *S. mutans* from mother to child include high *S. mutans* counts in the mother’s saliva and a diet rich in sucrose, and it has been suggested that the production of mutacin by the *S. mutans* strains harbored by the mother may also enhance this process.^85^ Transmission of *S. mutans* can be effectively reduced if mothers from a high‐caries‐risk population regularly use xylitol‐containing chewing gum, as concluded in a recent meta‐analysis.^86^

Both, predentate and dentate children are more likely to harbor not only *S. mutans*, but also *Streptococcus sobrinus*, *Actinomyces* species, *Campylobacter rectus*, *Fusobacterium nucleatum*, *Prevotella intermedia,* and *Porphyromonas gingivalis*, if their caregivers also carry these taxa.^87^ Similarly, detection of *Capnocytophaga gingivalis*, *Capnocytophaga ochraea*, *Capnocytophaga rectus,* and *Treponema denticola* between 3‐ to 9 year‐old children and their mothers was highly consistent.^88^ Concordance in colonization status with *P. gingivalis* has been observed among different family members, including fathers and their children.^89^ Intra‐familial transmission of *Prevotella*, between 23 mothers and their children of 2‐3 years of age, was found for *Prevotella nigrescens* and *Prevotella pallens,* but not for *P. intermedia*, and this transmission was more obvious in periodontally healthy mothers than in those with periodontal disease.^90^ A larger group of mother‐child pairs and a higher number of isolates should be assessed to investigate the reproducibility of these findings and to address the potential mechanisms behind the observed differences in transmissibility of *Prevotella* species.

One of the few longitudinal studies on oral microbiota of infants assessed salivary anaerobic bacteria from 44 infants during their first year of life, at 2, 6, and 12 months of age.^91^ *Veillonella* species and *Prevotella melaninogenica* (both obligate anaerobes), as well as facultatively anaerobic *Actinomyces* species were frequently present in the earliest samples taken, when infants were just 2 months old, and the frequency of *F. nucleatum*, nonpigmented *Prevotella* species, *Porphyromonas catoniae,* and *Leptotrichia* species increased remarkably between 2 and 6 months of age.^91^ *Actinobacillus* (now *Aggregatibacter*) *actinomycetemcomitans*, *P. gingivalis,* and *Bacteroides forsythus* (now *Tannerella forsythia*) were not isolated at any time point. Large interindividual differences in the complexity of the anaerobic microbiota among the infants were observed (eg, 0‐8 anaerobic species were isolated from saliva of 2‐month‐old infants, all still predentate). These findings support the studies above, in which caregivers were compared with their infants, and strengthen the notion that not the presence of the teeth as such but rather exposure to the various species of bacteria from family members determines the timing of the acquisition of anaerobes at an early age.^91^ At the clonal level, the turnover rate of some bacterial taxa (eg, *P. melaninogenica*) is shown to be high in children compared with adults, while clones of other taxa (eg, *A. actinomycetemcomitans*) remained very stable, once colonizing the individual.^92^ These interesting, yet quite limited, findings make us realize that it will not be possible to elucidate the complexity of microbial acquisition at an individual level in the absence of large‐scale detailed longitudinal studies in which microbial profile analyses from both children and their direct caregivers (including assessment of strain transmission and colonization persistence and genetic background) are performed, as discussed later, under heritability of the oral microbiome.

### 4.4 Feeding habits and other behaviors during infancy

The mammalian nature of humans provides the best food for their infants—breast milk. Breast milk contains the optimal nutrients—complex proteins, lipids and carbohydrates, as well as numerous bioactive factors including antimicrobial enzymes, antibodies, immune cells, microbiota and even stem cells—to promote healthy development of the child.^93^, ^94^, ^95^, ^96^ To match the needs of the infant, the composition of the breast milk changes during a single feed and throughout different phases of the lactation period.^93^ The first milk produced, the colostrum, is compositionally very different from mature milk: it is high in whey protein, secretory immunoglobulins, human milk oligosaccharides, and leukocytes, and very low in casein, lactose, and fat compared with mature milk.^93^, ^96^ This suggests that the primary role of colostrum is immunologic and that of mature milk is nutritional.

The immune system of a newborn does not function properly: it has incomplete physical and chemical barriers, poor innate immune cell function, limited and delayed secretory IgA production, incomplete complement cascade function, and insufficient anti‐inflammatory mechanisms of the respiratory and gastrointestinal tracts.^95^ Breast milk, with its antimicrobial properties (eg, lactoferrin and lysozyme) and active and passive immunity components (eg, leucocytes, secretory immunoglobulins), thereby functions as a gatekeeper until the infant’s own immune system matures. For instance, among infants admitted to hospital intensive care, an inverse relationship was found between the concentrations of the defense proteins—dermcidin and lysozyme—in the breast milk of the mother and oral yeast colonization status in her child.^97^

Over a decade ago, the first reports on bacteria as a natural component of breast milk appeared. The sources of bacteria in breast milk are suggested to be both extrinsic (from the skin surface of the mother and her infant, and from the oral cavity of the infant)^98^ as well as intrinsic (from the mother) via the entero‐mammary pathway.^99^ It has been estimated that breast milk contains about 10^6^ bacterial cells per mL,^100^ the most predominant genera being *Streptococcus*, *Staphylococcus*, *Bifidobacterium*, *Propionibacterium,* and *Lactobacillus*.^101^ Different factors, including maternal body mass index, delivery mode, and duration of lactation, have been reported to affect the species of microbes present in breast milk.^27^ Several studies have demonstrated the transfer of specific bacterial strains from a mother to her infant via breast milk,^99^ suggesting that the natural role of breast microbiota is to seed the gastrointestinal tract of the infant. The role of human milk oligosaccharides, the main carbohydrates of the breast milk and indigestible by the infant, is to provide nutrients for these newly acquired microbiota.^93^

It has been demonstrated that, during breastfeeding, infant saliva reacts with the breast milk and produces reactive oxygen species.^102^ Saliva of neonates contains high levels of purine metabolites (xanthine, hypoxanthine, adenosine, inosine, and guanosine), which gradually decrease with time, reaching the levels found in adults when the infant is about 6 months of age. Xanthine and hypoxanthine are substrates for xanthine oxidase, an enzyme strongly expressed in breast milk and, together with the enzyme lactoperoxidase, are involved in hydrogen peroxide production. This suggests a unique biochemical synergism between the infant saliva and the breast milk components that could shape the oral and gut microbiota.^102^

The World Health Organization and the United Nations Children’s Fund recommend initiation of breastfeeding within an hour of birth and exclusive breastfeeding for the first 6 months of life.^103^ Sometimes, however, breastfeeding is impaired or impossible, and industrially produced infant formula, based on cow’s milk or soya milk and prepared from powder or liquid, is used instead, as a breast milk substitute. Although attempts are made by the manufacturers to mimic the composition of breast milk as closely as possible (eg, by supplementing the formula with iron, nucleotides, various mixtures of lipids and fatty acids, probiotics, and other compounds),^104^ the versatility of natural breast milk, individually tailored for each infant, will never be mimicked in a factory.

Knowing the striking differences between the 2 nutrient sources—breast milk and formula milk—it is not surprising to find differences in the oral microbiota of infants fed by these different modes. For instance, buccal mucosal swabs from 4‐ to 8‐week‐old formula‐fed infants showed a higher proportion of species from the phylum Bacteroidetes, especially those of the genus *Prevotella*, than breastfed infants.^105^ The microbial profiles of oral swabs of 3‐month‐old infants, exclusively breastfed, differed significantly from those who were formula‐fed.^106^ The number of species of bacteria detected was significantly lower in the breastfed infants, and they had a higher prevalence of *Actinomyces gerencseriae* and *Streptococcus australis*, compared with formula‐fed infants, who were more frequently colonized by *Prevotella*, *Lautropia mirabilis*, *Neisseria*, TM7, *Kingella*, *Granulicatella*, *Leptotrichia*, *Solobacterium moorei*, *Haemophilus,* and *Veillonella*.^106^ The same group found that lactobacilli colonized the oral cavity of 4‐month‐old breastfed infants significantly more frequently than that of formula‐fed infants.^107^ The dominant *Lactobacillus* species was *Lactobacillus gasseri*, which was detected at higher levels in breastfed infants than in formula‐fed infants and displayed probiotic traits, such as inhibition of several caries and periodontal disease‐associated taxa in vitro.^107^ A recent study found that bacterial diversity of saliva was still lower in 2‐year‐old children who were breastfed for 12 months than in those who were breastfed for <6 months, and that salivary microbial profiles of these 2 groups differed, even at the age of 7 years.^41^ These findings suggest that initial differences in the feeding mode, originally influencing the acquisition of the microbiota, might actually have long‐lasting consequences on the oral microbiota of the child.

A recent study followed acquisition of the salivary microbiome in 9 infants from birth until 12 months of age at monthly intervals and concluded that this process occurs in an organized pattern.^108^ Interestingly, the transition from liquid to solid food, rather than eruption of the teeth, seemed to introduce major compositional changes in the microbiome.

If infants are exposed to sweetened liquids during the bottle‐feeding period or to sugar‐containing foods and snacks once they consume solid foods, this becomes a major ecological factor in driving changes in the oral microbial communities. Natural nutrients for oral microbiota are salivary glycoproteins, the degradation of which requires complex microbial interactions.^109^ As a result of frequent acidification of the environment by microbial metabolism of sucrose or other fermentable sugars, microbial diversity is lost and aciduric and acidogenic taxa are enriched—the microbial changes frequently observed in infants and children with early childhood caries.^110^ This topic will not be discussed further here because it goes beyond development of a healthy oral microbiome and has recently been reviewed.^111^

Besides feeding habits, the use of a pacifier has been shown to affect the oral microbiota of infants. Its use has been associated with higher salivary levels of lactobacilli and yeasts.^112^, ^113^, ^114^ Significant relationships were found between recovery of yeasts, the use of a pacifier in infants over 12 months of age, and the mother cleaning the child’s pacifier in her own mouth.^115^ Interestingly, pacifier‐cleaning habits (boiling the pacifier versus the parent sucking it) have been shown to affect the salivary bacterial profiles of infants and, if parents regularly sucked the pacifier instead of boiling it, their children had a reduced risk for allergy development at an older age.^116^

As already mentioned previously, medication use influences the microbiome. The effect of antibiotics in the first 2 years of life on oral microbiota later in life was recently assessed in saliva of 90 Swedish children who were followed‐up until 7 years of age.^41^ Of the 90 children included, 30% had received antibiotics (amoxicillin or phenoxymethylpenicillin) in the first year of life and 44% had received those antibiotics in the second year of life, mainly to treat early otitis media. The authors found that the genera *Prevotella* and *Actinomyces*, and species belonging to the genera *Fusobacterium*, *Veillonella*, and *Lactobacillus*, were present in saliva at higher levels in 7‐year old children who were exposed to antibiotics early in life, whereas *Neisseria* and *Streptococcus mitis/dentisani* were elevated in subjects not exposed to antibiotics as infants.^41^

Predentate infants with mothers who smoked had significantly different oral microbial profiles compared with those of infants with mothers who did not smoke, and exhibited higher proportions of *F. nucleatum* and *Campylobacter concisus*.^57^ As the oral microbial communities of smokers have been shown to be different from those of nonsmokers and are enriched for anaerobes,^117^, ^118^ it is not surprising that infants have acquired these taxa.

One behavioral aspect that certainly influences the oral microbial communities at any age, is the level of oral hygiene. The age when parents started brushing the teeth of their children correlated with caries prevalence in 5‐ to 8‐year‐old children,^119^ supporting the advice to start toothbrushing as soon as the first tooth begins to erupt. Poor oral hygiene was associated with a more diverse salivary microbiome, with reduced levels of streptococci and increased species of the genus *Veillonella* in 7‐ to 15‐year‐old Thai children,^120^ and with a more diverse supragingival plaque microbiome, enriched in the genera *Corynebacterium*, *Leptotrichia, Porphyromonas,* and *Selenomonas*, and depleted in the genera *Neisseria, Actinomyces*, *Streptococcus*, and *Rothia*, in 6‐ to 14‐year‐old Dutch children.^121^

Taken together, not only the immune system and general health of infants, but also their oral microbiota, are shaped by exposure to the chemical, immunological, and microbiological components of breast milk. In addition, many other behavioral habits, such as diet, oral hygiene, and pacifier use, and also exposure to antibiotics early in life and maternal smoking, influence the oral microbiota that becomes established in the child.

### 4.5 Heritability of the oral microbiome

Symbiotic coevolution of the host with its microbes makes it plausible that human genes (genome) may shape microbial communities toward those that are most beneficial for the individual host. There is indeed an increasing body of evidence that, besides environmental factors, such as diet and microbial exposure, the genetic background of the host influences the microbiome.^122^ A pioneering study, which set the stage for studies on the role of the genome of the host in shaping the oral microbiota, was performed 60 years ago and found significant heritability for salivary streptococci and unclassified bacteria, as well as for different salivary properties, in 14‐ to 38‐year‐old twin pairs.^123^ The second study on this subject appeared 4 decades later, in which colonization with *S. mutans* was found to be significantly heritable in 3‐year‐old Brazilian twins, where 52% of colonization was heritable and 48% attributable to environmental factors.^124^ The same group assessed the levels of 82 different species of oral bacteria in plaque from primary dentition of monozygotic and dizygotic twin pairs, with and without caries.^125^ Besides moderate (47%) heritability for *S. mutans* in the caries group, high (80%) heritability was reported for *Gemella morbillorum* and moderate‐to‐high heritability (56%‐60%) for *Abiotrophia defectiva*, *Gemella haemolysans*, and streptococcal species, all associated with caries‐free children.

Several Australian twin cohorts have been set up to study tooth emergence and oral health.^126^ Using a monozygotic twin model, colonization with *S. mutans* in 151 monozygotic twin pairs was assessed from birth, every 3 months, until 3 contiguous positive scores for both twins were obtained. The mean age of colonization was 12.7 (standard deviation: 6.1) months, with the earliest colonization at 2.4 months and the latest being at just over 2.5 years.^79^ The concordance for colonization with *S. mutans* was low in these children. In a classical twin study model of the same cohort, no difference was found in concordance for colonization with *S. mutans* or lactobacilli between monozygotic and dizygotic twins,^127^ leading the authors to conclude that environmental or epigenetic factors may affect colonization with *S. mutans* and lactobacilli more than the genetic background of the individual.

A recent publication on 2 Australian twin cohorts in which the supragingival plaque microbiome of over 240 monozygotic and dizygotic twin pairs, 5‐11 years of age, was compared, reported higher similarity in microbial profiles of the monozygotic twins than of the dizygotic twins.^128^ None of the heritable taxa was associated with caries but decreased with age, and the most heritable were *Prevotella pallens*, uncultured *Veillonella* species, unclassified *Pasteurellaceae*, and *Corynebacterium durum*. In line with the ecological plaque hypothesis,^129^ the taxa associated with dental caries (including *S. mutans*) were not heritable but driven by environmental factors, such as frequency of sucrose consumption.

Another recent, largest‐to‐date twin salivary microbiome study (*N* = 752 twin pairs, 11‐24 years of age, US) found that heritability extended across nearly all observed taxa except fusobacteria.^130^ By comparing, using genome‐wide association analysis, the salivary microbiome and the genetic variants in 1480 individuals unrelated to the twins and to each other, individual loci in chromosomes 7 and 12 that were significantly associated with different microbiome phenotypes were identified.

In contrast to the findings above, a longitudinal study (*N* = 45 twin pairs, followed for over 10 years, US) failed to find any difference in similarity between the salivary microbiomes of the monozygotic and dizygotic twin pairs,^131^ which could be a result of the much smaller sample size of this study than the studies by Gomez et al^128^ and Demmitt et al.^130^ Similarly, another even smaller twin study (*N* = 16 twin pairs) failed to find a difference in subgingival microbiomes between the monozygotic and dizygotic pairs.^132^ The role of shared environment was demonstrated by higher similarity between the microbial profiles of the co‐twins, irrespective of their zygosity, than between unrelated individuals,^131^, ^132^ and the fact that this similarity decreased significantly with age or when the twins no longer cohabited.^131^

It seems that, provided the study has a sufficient sample size, the genotype of the host influences the selection of the beneficial (health‐associated) microbial taxa, at least in relation to dental caries. Microbiome composition across different body sites, including the oral cavity, has been shown to correlate with genetic variation in immunity‐related pathways,^133^ implying that the taxa usually associated with gingival inflammation might also be influenced by the host genotype. An association between host human leukocyte antigen class II, tumor necrosis factor alpha genetic profile, and colonization of *S. mutans*, *Lactobacillus casei*, and *Lactobacillus acidophilus* has been demonstrated in African American women.^134^ The role of host factors in mother‐to‐child transmission of human papillomavirus was recently demonstrated by a study in which mother‐infant concordance in a certain human leukocyte antigen‐G genotype increased the risk of human papillomavirus positivity in the infant’s oral mucosa.^135^ Different salivary properties, important in maintaining oral health, also have a genetic component. For instance, several studies have demonstrated heritability of: salivary flow rate, pH, and amylase activity;^123^ salivary agglutinin activity, parotid flow rate, and total protein;^136^ lactoferrin and peroxidase;^137^ and sizeable heritable components for testosterone and estradiol concentrations in saliva of adolescents.^138^ The relationship among salivary enzymatic activities, salivary pH and microbial composition of saliva was demonstrated in young healthy adults.^139^ A recent study found a relationship between copy number variation in the salivary amylase gene, *AMY1*, and microbiome composition and function in both saliva and gut: the microbiomes of subjects with high copy numbers of *AMY1* had higher levels of salivary *Porphyromonas* species, while their gut microbiota had increased abundance of resistant starch‐degrading microbes, produced higher levels of short‐chain fatty acids, and drove higher adiposity when transferred to germ‐free mice.^140^ Taken together, different host‐determined salivary properties are involved in shaping the composition of both oral and gut microbiomes of the host.

In addition to the genetic factors, which are determined by the linear sequence of DNA (the genes), the phenotype can be altered as a result of epigenetic modifications of chromatin structure. These include DNA methylation and covalent modifications of proteins that bind DNA, resulting in altered gene activity and expression.^141^ Maternal smoking, delivery mode, exposures to medications early in life, and most probably also the first encounters with microbiota, are only a few of the epigenetic factors that may affect the individual phenotype and should be the focus of future studies.

### 4.6 Microbial changes with age: Transition from childhood to adolescence

At around the age of 6 years, the primary teeth start to exfoliate and are replaced by the permanent teeth. This period, during which both primary and permanent teeth are present, is called the mixed‐dentition period. Eruption of the permanent dentition begins with the mandibular central incisors and the first mandibular and maxillary molars and is usually complete at around the age of 12, with eruption of the second molars.^142^ During the mixed‐dentition period, the oral ecosystem undergoes various changes related to the eruption of permanent teeth as well as caused by the onset of puberty.

When microbial profiles of plaque from children with primary, mixed, and permanent dentition are compared, supragingival plaque from the permanent dentition has higher levels of both gram‐positive and gram‐negative anaerobic species than the primary dentition. Subgingival plaque from the mixed dentition is in the stage of greatest transition regarding microbial composition and has a significantly higher proportion of gram‐negative anaerobes than subgingival plaque from either primary or permanent dentition.^57^ Several other studies, using targeted microbiological approaches, also report an increase in anaerobic taxa with transition to the mixed‐dentition phase.^143^, ^144^, ^145^, ^146^, ^147^, ^148^ As occlusion is not complete, dental plaque and food remnants stagnate at the occlusal surfaces of partially erupted permanent molars.^149^ The gingival swelling around the erupting teeth may interfere with oral hygiene measures, explaining the finding of more mature dental plaque, while reduced tissue resistance around the erupting teeth may increase gingival bleeding. Indeed, preschool children with primary dentition are shown to have low susceptibility to gingivitis, which gradually increases at the age of 6‐8 years with the eruption of the first permanent teeth and may become aggravated around 10‐13 years of age,^150^ coinciding with increased hormonal activity around puberty.

During puberty, 2 hormonal cascades, released by the hypothalamic‐pituitary‐gonadal axis and the hypothalamic‐pituitary‐adrenal axis, play critical roles in the development of important body systems, including the reproductive and immune systems. The hypothalamic‐pituitary‐gonadal axis controls development by secretion of gonadotropin‐releasing hormone: this leads to the release of luteinizing hormone and follicle‐stimulating hormone, which promote the production of testosterone and progesterone in female gonads and the production of testosterone in male gonads.^151^, ^152^ In both male and female individuals testosterone is converted to estradiol, which has high circulating levels and mediates growth spurts and sexual changes during puberty.^153^ The hypothalamic‐pituitary‐adrenal axis is involved in the response to physiological or psychological stressful stimuli by releasing stress hormones, such as cortisol, to help individuals cope with stressful events; responses include mobilization of energy stores, reduction of inflammation, and enhanced immune activity.^154^, ^155^ This is reflected in increased cortisol levels in saliva during puberty.^156^, ^157^

Mombelli and colleagues assessed changes in the subgingival microbiota throughout puberty in 42 children (20 girls) from the age of 11, every 4‐5 months for 4 years, and compared these changes with the oral clinical status and pubertal maturation of the children.^158^, ^159^, ^160^ The gingival bleeding and total bacterial counts increased at the onset of puberty and decreased after the age of 14 years. The prevalence of *A. odontolyticus* and *Capnocytophaga* species increased with time. In boys, the initial pubertal phase was associated with increases in gingival bleeding and in the prevalence of *Bacteriodes* species (now *P. intermedia* and *P. melaninogenica),* while in girls puberty started with increases in gingival bleeding and in the prevalence of *A odontolyticus*. Gingival bleeding was preceded by an increase in *Capnocytophaga* species in the subgingival plaque, while the extent of the bleeding correlated positively with the prevalence and proportion of spirochetes, *Eikenella corrodens,* and negatively ‐ with the proportion of *Actinomyces viscosus*. The increase in frequency of black pigmented *Bacteriodes* (*P. intermedia*) was secondary to the increase in gingival bleeding. This study, among others on this topic, suggests that the changes in subgingival microbiota during puberty are caused by increased gingival inflammation,^153^ and removal of plaque by oral hygiene is key to manage puberty‐associated gingivitis.^161^

The effects of sex hormones on other microbial niches, such as human vaginal microbiota^162^ and rodent gut,^163^ have recently been demonstrated. Although the relationship with the oral microbiota has so far not been directly shown in clinical studies, exposure of individual microbiota to estradiol or to stress hormones does affect their growth and/or virulence properties in vitro. For instance, the growth of single cultures of *P. intermedia* and *P. melaninogenica* was enhanced when menadione (vitamin K), necessary for their growth, was replaced with estradiol in the growth medium,^164^ as was the growth and virulence of *C. rectus*.^165^ Additionally, estradiol was shown to impair the T‐helper 17 immune response mounted against *Candida albicans*.^166^ When the presence of stress hormones—catecholamines and cortisol—was tested on selected periodontitis‐associated taxa in vitro,^167^ none of the hormones affected the growth of *P. gingivalis*, whereas growth of *P. intermedia* and *E. corrodens* was inhibited and the growth of *F. nucleatum* and *T. forsythia* was enhanced. Interestingly, ex vivo exposure of dental plaque from a periodontally healthy individual to cortisol for 2 hours resulted in changes in microbial activity, mainly enhancing the metabolism of *F. nucleatum* and *Leptotrichia goodfellowii*,^168^ suggesting that exposure to cortisol resembles the community‐wide expression profiles observed in periodontitis and its progression in vivo. In a recent study, salivary cortisol levels in 59 Swedish children at 6, 12, and 24 months of age were compared with salivary microbial composition, and an inverse relationship between cortisol and the most common taxon (determined using operational taxonomic units), classified to the family Streptococcaceae, was observed.^169^ Taken together, the microbiota is able to respond to changes in hormonal levels.

Other factors, besides physiological changes during the mixed‐dentition stage and puberty, are known to influence the composition of the oral microbiota during adolescence; these include changes in behavioral habits, such as increased frequency of intake of sugar‐containing snacks and beverages, smoking, alcohol consumption,^170^, ^171^, ^172^ and orthodontic treatment with fixed appliances^173^, ^174^, ^175^

### 4.7 Beyond bacteria—acquisition and establishment of other microbes

Besides bacteria, other microbes, such as fungi, Archaea, viruses, the newly classified candidate phyla radiation group of ultra‐small bacteria and even protozoa, are part of the oral microbiome.^176^, ^177^ There is an increasing body of evidence that this “dark matter” of the human microbiome has important functions and contributes to shaping and maintaining the microbiome and the immune system of the host.^177^ As a result of difficulties in their isolation, culture, and identification, the majority, however, is still poorly described.

In the oral cavity, very few studies have looked beyond the bacterial component, with fungal studies being the most commonly studied from the list of the “forgotten” microbes. Several culture studies have addressed vertical transmission of fungi from mother to infant. Infants delivered vaginally to mothers with vaginal colonization by *Candida* had a higher prevalence of oral *Candida* than infants delivered by Cesarian section or infants delivered vaginally to mothers without vaginal *Candida* colonization.^178^, ^179^ A recent systematic review on this topic concludes that vaginal delivery appears to promote oral yeast colonization in early life, and that there is no difference between breastfed and bottle‐fed infants.^180^ During the first year of life, the oral cavity of 15% of 100 Swedish infants was colonized by *Candida* and its colonization at the age of 6 months correlated negatively with the concentration of lactoferrin in breast milk.^181^

Based on a traditional culture approach, *C. albicans* and *Candida parapsilosis* have been identified as the most prevalent *Candida* species in infants^114^ and in older children.^182^ Interestingly, in a study on 53 newborns in Chile, an oral *Candida glabrata* infected with intracellular *Helicobacter pylori* was isolated from a newborn,^183^ suggesting a potential fungal role in mother‐infant transmission of microbes.

A recent study on the entire fungal community (mycobiome) of skin, oral, and anal swabs of infants (*N* = 17; 7 born vaginally) from birth over the first 30 days of life found that each body site harbored site‐specific mycobiomes that were highly variable over the first month.^184^ The oral mycobiome was significantly less diverse than the skin or the anus, with the most prevalent or abundant oral taxa being *C. parapsilosis* (96% of samples, average 25% of reads), *Candida tropicalis* (89% of samples, 15% of reads), *Saccharomyces cerevisiae* (64% of samples, 11% of reads), *Candida orthopsilosis* (63% of samples, 10% of reads), *C. albicans* (57% of samples), and *Cladosporium velox* (8% of reads). The individual mycobiome was often dominated by 1 specific taxon. The fungal communities remained highly variable in time, and did not increase in diversity during the first month of life. There was no difference in the composition of the oral mycobiome according to delivery mode, apart from finding a higher proportion of *C. orthopsilosis* after Cesarian section.^184^ Another recent mycobiome study, comparing dental plaque from 17 children (7‐10 years old), with and without caries, found higher fungal diversity in caries‐free children, with the most prevalent taxa being *Saccharomyces*, *C. albicans*, *Naganishia diffluens,* and *Rhodotorula mucilaginosa*,^185^ indicating that there is still much to be discovered with respect to the oral mycobiome. Reports on fungal involvement in shaping human innate and adaptive immunity^186^ highlight the need for studies on the early acquisition and establishment of oral fungal communities.

Viruses, including both eukaryotic and prokaryotic viruses found in and on the human body, are collectively called the virome.^187^ When samples from different body habitats of 102 healthy adults, participating in the Human Microbiome Project, were analyzed for eukaryotic DNA viruses, each individual appeared to have unique viral fingerprints at different body sites.^188^ In the oral cavity, the most prevalent eukaryotic DNA virus was *Roseolovirus* (97% of subjects), followed by *Lymphocryptovirus* (29%), *Betapapillomavirus* (21%), *Mastadenovirus* (18%), and *Polyomavirus* (15%).^188^ A study on the entire virome (both eukaryotic viruses and bacteriophages [viruses infecting bacteria]), in saliva of 21 adults, found only a few homologues to viruses of eukaryotes (herpesviruses and circoviruses), with the vast majority being bacteriophages.^189^ In most of the subjects, the virome consisted predominantly of viruses specific for the bacterial phyla Proteobacteria, Firmicutes, and Actinobacteria. Additionally, the finding of viral homologues in study participants who shared the same household led the authors to propose that sharing the same environment has a role in shaping salivary viral ecology.^189^ The salivary virome of 11 unrelated adults, repeatedly sampled over 60 days, appeared to be highly stable, and consisted predominantly of bacteriophages.^190^ No studies to date have assessed the oral virome in children. A recent study on the delivery mode and virome found a strong correlation between vaginal birth mode and diversity and composition of the gut virome in children at 1 year of age.^191^

Although the role of bacteriophages in the oral cavity remains unclear, it is highly likely that they contribute to shaping the bacterial community.^192^, ^193^ Their relationship with oral health was demonstrated by Wang et al,^194^ who associated cross‐infective phages (phages that could infect different bacterial taxa besides their putative bacterial host) with commensal bacteria and periodontal health and the absence of these phages with periodontal disease. How bacteriophages are acquired and transmitted, and their actual role in the oral ecosystem, remain to be established.

Archaea, genetically distinct from bacteria and eukaryotes, also called the third domain of life, are methanogens that utilize hydrogen to reduce carbon dioxide, acetate, and a variety of methyl compounds into methane.^195^, ^196^ No studies have assessed archaeal oral colonization in infants, while studies in adults find these anaerobic microbes in subgingival plaque of periodontally healthy individuals and report their increase in prevalence and abundance with periodontal disease.^195^ The fact that these microbes are acquired early in life was demonstrated by finding *Methanobrevibacter smithii* in gastric juice of newborns. Their abundance was associated with breastfeeding, suggesting transmission from mother via breast milk.^197^ Additionally, when methane was measured in the exhaled air in 112 volunteers aged 1‐80 years, exhaled methane levels were higher than the inhaled air levels in all cases, including the children.^198^

Recently, the tree of life has been expanded with yet another group of microbes—ultra‐small bacteria, classified as the candidate phyla radiation group.^177^ The candidate phyla radiation is a major bacterial lineage or superphylum, containing over 70 phyla, 3 of which are detected within the human oral microbiome: Saccharibacteria (formerly TM7), SR1, and GN02; Saccharibacteria is the most abundant phylum and associated with gingivitis and periodontitis.^199^ The common feature of these microbes is their small genome and cell size and restricted metabolic capacities. So far, only a single candidate phyla radiation representative—Saccharibacteria member, *Nanosynbacter lyticus* strain TM7x—has been cultivated.^200^ The experiments with this first isolate have revealed that it is an obligate parasitic bacterial epibiont and needs to infect another bacterial species, such as *A. odontolyticus*, to survive. It affects the morphology and physiology of the host bacterium, suppresses the expression of tumor necrosis factor alpha in macrophages, and is potentially able to modulate the normal host immune response.^199^, ^200^ Again, as with viruses and archaea, there are no studies to date that address the colonization of infants with this group of microbes. Most likely, members of the oral candidate phyla radiation group are acquired together with their bacterial hosts via vertical and horizontal transmission. The role of the candidate phyla radiation group in shaping the oral microbiome and their interplay with other microbial members is yet another exciting field to be studied.

Among the single‐celled eukaryotes or protozoa, *Entamoeba gingivalis* has been associated with periodontal disease in adults,^201^ and is presumably acquired through contact with water and contaminated foods. A single study, in which oral carriage of *E. gingivalis* in children (*N* = 154, 2‐18 years of age) was assessed, found higher *E. gingivalis* counts among Polish children from urban areas than from rural areas; the authors did not report the actual prevalence and abundance data of *E. gingivalis* or the relationship with the age of children.^202^ A recent longitudinal study from Luxembourg, in which colonization of infant gut with bacteria, protozoa, fungi, and Archaea was followed in 15 infants from birth for 12 months, showed that all these types of microbes could be found in infant feces, although colonization with protozoa was not stable throughout the first year of life.^203^

In summary, the role of microbes other than the bacterial component of the microbiome on the establishment and maintenance of a healthy oral microbial ecosystem is highly understudied. A holistic view on microbial interactions will certainly bring valuable insights into how the equilibrium among all the different members of the oral ecosystem is established and preserved, and how that contributes to the wellbeing of the human host.

## 5 CONCLUDING REMARKS

Acquisition and establishment of a healthy microbiota is of great importance for a symbiotic host‐microbiota relationship. Early exposure to certain microbes seems pivotal in development of a healthy immune system, and may even have long‐term consequences on general health. Based on the current literature, we can conclude that the oral microbial ecosystem is shaped in utero, by, most likely, exposure of maternal antigens to the immune system of the infant, in this way preparing the infant for postnatal microbial encounters. This makes a healthy oral ecosystem of the mother of paramount importance. The modes of birth and feeding, as well as perinatal exposure to medications, determine which microbes will be encountered first and if at all. Teeth eruption and oral hygiene habits, together with sugar consumption, exposure to antibiotics, maternal smoking, and oral health status of the caregiver affect the developmental trajectories of infants’ and childrens’ oral microbiota. Besides these environmental factors, there is a growing body of evidence that the health‐associated oral microbiota has a heritable component.

This review has also identified a number of outstanding questions for future research. To name just a few: What are the mechanisms of mother‐fetus microbiota crosstalk? Is this crosstalk selective to certain groups of microbiota? The oral cavity of infants has not been as excessively sampled as that of adults and longitudinal studies on multiple oral niches are required. Although several methods have been used to collect oral samples from infants (eg, saliva, buccal mucosa, tongue, or alveolar ridge swabs), it remains unclear which method, or which combination of methods, would give the most informative results. To elucidate the complexity of the microbial acquisition at an individual level, large‐scale detailed longitudinal studies, in which microbial profile analyses of both children and their direct caregivers are performed, and which also include a holistic assessment of all microbiota, investigate the transmission and colonization persistence of strains, and determine the genetic and epigenetic backgrounds of the host, will be required.

## References

[1] Blaser MJ , Cardon ZG , Cho MK , et al. Toward a predictive understanding of earth’s microbiomes to address 21st century challenges. mBio. 2016;7(3):e00714‐e00716. 27178263 10.1128/mBio.00714-16 PMC4895116

[2] McFall‐Ngai M , Hadfield MG , Bosch TCG , et al. Animals in a bacterial world, a new imperative for the life sciences. Proc Natl Acad Sci USA. 2013;110(9):3229‐3236. 23391737 10.1073/pnas.1218525110 PMC3587249

[3] Sender R , Fuchs S , Milo R . Are we really vastly outnumbered? Revisiting the ratio of bacterial to host cells in humans. Cell. 2016;164(3):337‐340. 26824647 10.1016/j.cell.2016.01.013

[4] Cox Laura M , Blaser MJ . Pathways in microbe‐induced obesity. Cell Metab. 2013;17(6):883‐894. 23747247 10.1016/j.cmet.2013.05.004 PMC3727904

[5] Kilian M , Chapple ILC , Hannig M , et al. The oral microbiome – an update for oral healthcare professionals. BDJ. 2016;221(10):657. 27857087 10.1038/sj.bdj.2016.865

[6] Huttenhower C , Gevers D , Knight R , et al. Structure, function and diversity of the healthy human microbiome. Nature. 2012;486:207‐214. 22699609 10.1038/nature11234 PMC3564958

[7] Daalderop LA , Wieland BV , Tomsin K , et al. Periodontal disease and pregnancy outcomes: overview of systematic reviews. JDR Clin Trans Res. 2018;3(1):10‐27. 30370334 10.1177/2380084417731097 PMC6191679

[8] Iheozor‐Ejiofor Z , Middleton P , Esposito M , Glenny AM . Treating periodontal disease for preventing adverse birth outcomes in pregnant women. Cochrane Database Syst Rev. 2017.6(6):CD005297. 28605006 10.1002/14651858.CD005297.pub3 PMC6481493

[9] Madianos PN , Bobetsis YA , Offenbacher S . Adverse pregnancy outcomes (APOs) and periodontal disease: pathogenic mechanisms. J Periodontol. 2013;84(4 Suppl):S170‐S180. 23631577 10.1902/jop.2013.1340015

[10] Pelzer E , Gomez‐Arango LF , Barrett HL , Nitert MD . Review: maternal health and the placental microbiome. Placenta. 2017;54:30‐37. 28034467 10.1016/j.placenta.2016.12.003

[11] Perez‐Munoz ME , Arrieta MC , Ramer‐Tait AE , Walter J . A critical assessment of the "sterile womb" and "in utero colonization" hypotheses: implications for research on the pioneer infant microbiome. Microbiome. 2017;5(1):48. 28454555 10.1186/s40168-017-0268-4 PMC5410102

[12] Aagaard K , Ma J , Antony KM , Ganu R , Petrosino J , Versalovic J . The placenta harbors a unique microbiome. Sci Transl Med. 2014;6(237):237ra65. 10.1126/scitranslmed.3008599 PMC4929217 24848255

[13] Macpherson AJ , Uhr T . Induction of protective IgA by intestinal dendritic cells carrying commensal bacteria. Science. 2004;303(5664):1662‐1665. 15016999 10.1126/science.1091334

[14] Bright M , Bulgheresi S . A complex journey: transmission of microbial symbionts. Nat Rev Microbiol. 2010;8(3):218‐230. 20157340 10.1038/nrmicro2262 PMC2967712

[15] Jeurink PV , van Bergenhenegouwen J , Jimenez E , et al. Human milk: a source of more life than we imagine. Benef Microbes. 2013;4(1):17‐30. 23271066 10.3920/BM2012.0040

[16] Gilbert SF . A holobiont birth narrative: the epigenetic transmission of the human microbiome. Front Genet. 2014;5:282. 25191338 10.3389/fgene.2014.00282 PMC4137224

[17] Dominguez‐Bello MG , Costello EK , Contreras M , et al. Delivery mode shapes the acquisition and structure of the initial microbiota across multiple body habitats in newborns. Proc Natl Acad Sci USA. 2010;107(26):11971‐11975. 20566857 10.1073/pnas.1002601107 PMC2900693

[18] Zaura E , Nicu EA , Krom BP , Keijser BJ . Acquiring and maintaining a normal oral microbiome: current perspective. Front Cell Infect Microbiol. 2014;4(85). 10.3389/fcimb.2014.00085 PMC4071637 25019064

[19] Niederman R . Pregnancy gingivitis and causal inference. Evid Based Dent. 2013;14(4):107‐108. 24357820 10.1038/sj.ebd.6400966

[20] Mold JE , Michaelsson J , Burt TD , et al. Maternal alloantigens promote the development of tolerogenic fetal regulatory T cells in utero. Science. 2008;322(5907):1562‐1565. 19056990 10.1126/science.1164511 PMC2648820

[21] Gomez de Aguero M , Ganal‐Vonarburg SC , Fuhrer T , et al. The maternal microbiota drives early postnatal innate immune development. Science. 2016;351(6279):1296‐1302. 26989247 10.1126/science.aad2571

[22] Hillman NH , Kallapur SG , Jobe AH . Physiology of transition from intrauterine to extrauterine life. Clin Perinatol. 2012;39(4):769‐783. 23164177 10.1016/j.clp.2012.09.009 PMC3504352

[23] Hyde MJ , Mostyn A , Modi N , Kemp PR . The health implications of birth by Caesarean section. Biol Rev Camb Philos Soc. 2012;87(1):229‐243. 21815988 10.1111/j.1469-185X.2011.00195.x

[24] Lagercrantz H . Stress, arousal, and gene activation at birth. New Physiol Sci. 1996;11(5):214‐218.

[25] Simsek Y , Karabiyik P , Polat K , Duran Z , Polat A . Mode of delivery changes oxidative and antioxidative properties of human milk: a prospective controlled clinical investigation. J Matern Fetal Neonatal Med. 2015;28(6):734‐738. 24903065 10.3109/14767058.2014.932345

[26] Dizdar EA , Sari FN , Degirmencioglu H , et al. Effect of mode of delivery on macronutrient content of breast milk. J Matern Fetal Neonatal Med. 2014;27(11):1099‐1102. 24107128 10.3109/14767058.2013.850486

[27] Cabrera‐Rubio R , Collado MC , Laitinen K , Salminen S , Isolauri E , Mira A . The human milk microbiome changes over lactation and is shaped by maternal weight and mode of delivery. Am J Clin Nutr. 2012;96(3):544‐551. 22836031 10.3945/ajcn.112.037382

[28] Toscano M , De Grandi R , Peroni DG , et al. Impact of delivery mode on the colostrum microbiota composition. BMC Microbiol. 2017;17(1):205. 28946864 10.1186/s12866-017-1109-0 PMC5613475

[29] Affolter M , Garcia‐Rodenas CL , Vinyes‐Pares G , et al. Temporal changes of protein composition in breast milk of Chinese urban mothers and impact of caesarean section delivery. Nutrients. 2016;8(8):504. 10.3390/nu8080504 PMC4997417 27548208

[30] Boustedt K , Roswall J , Dahlen G , Dahlgren J , Twetman S . Salivary microflora and mode of delivery: a prospective case control study. BMC Oral Health. 2015;15(1):155. 26631057 10.1186/s12903-015-0142-3 PMC4668661

[31] Zimmermann P , Curtis N . Factors influencing the intestinal microbiome during the first year of life. Pediatr Infect Dis J. 2018;37(12):e315‐e335. 29746379 10.1097/INF.0000000000002103

[32] Dominguez‐Bello MG , De Jesus‐Laboy KM , Shen N , et al. Partial restoration of the microbiota of cesarean‐born infants via vaginal microbial transfer. Nat Med. 2016;22:250‐253. 26828196 10.1038/nm.4039 PMC5062956

[33] Gomez‐Arango LF , Barrett HL , McIntyre HD , Callaway LK , Morrison M , Dekker NM . Antibiotic treatment at delivery shapes the initial oral microbiome in neonates. Sci Rep. 2017;7:43481. 28240736 10.1038/srep43481 PMC5378909

[34] Smaill FM , Grivell RM . Antibiotic prophylaxis versus no prophylaxis for preventing infection after cesarean section. Cochrane Database Syst Rev. 2014;10:CD007482. 10.1002/14651858.CD007482.pub3 PMC8078551 25350672

[35] Li H , Chen S , Wu L , et al. The effects of perineal disinfection on infant's oral microflora after transvaginal examination during delivery. BMC Pregnancy Childbirth. 2019;19(1):213. 31234808 10.1186/s12884-019-2350-3 PMC6591937

[36] Lif Holgerson P , Harnevik L , Hernell O , Tanner AC , Johansson I . Mode of birth delivery affects oral microbiota in infants. J Dent Res. 2011;90(10):1183‐1188. 21828355 10.1177/0022034511418973 PMC3173012

[37] Nelun Barfod M , Magnusson K , Lexner MO , Blomqvist S , Dahlen G , Twetman S . Oral microflora in infants delivered vaginally and by caesarean section. Int J Paed Dent. 2011;21(6):401‐406. 10.1111/j.1365-263X.2011.01136.x 21702851

[38] Li Y , Caufield PW , Dasanayake AP , Wiener HW , Vermund SH . Mode of delivery and other maternal factors influence the acquisition of *Streptococcus mutans* in infants. J Dent Res. 2005;84:806‐811. 16109988 10.1177/154405910508400905

[39] Pattanaporn K , Saraithong P , Khongkhunthian S , et al. Mode of delivery, mutans streptococci colonization, and early childhood caries in three‐ to five‐year‐old Thai children. Community Dent Oral Epidemiol. 2013;41(3):212‐223. 23106389 10.1111/cdoe.12013 PMC4418184

[40] Hurley E , Mullins D , Barrett MP , et al. The microbiota of the mother at birth and its influence on the emerging infant oral microbiota from birth to 1 year of age: a cohort study. J Oral Microbiol. 2019;11(1):1599652. 32128038 10.1080/20002297.2019.1599652 PMC7034431

[41] Dzidic M , Collado MC , Abrahamsson T , et al. Oral microbiome development during childhood: an ecological succession influenced by postnatal factors and associated with tooth decay. ISME J. 2018;12(9):2292‐2306. 29899505 10.1038/s41396-018-0204-z PMC6092374

[42] Ubeja RG , Bhat C . Mode of delivery and its influence on the acquisition of *Streptococcus mutans* in infants. Int J Clin Pediatr Dent. 2016;9(4):326‐329. 28127164 10.5005/jp-journals-10005-1386 PMC5233699

[43] Betran AP , Torloni MR , Zhang J , et al. What is the optimal rate of caesarean section at population level? A systematic review of ecologic studies. Reprod Health. 2015;12(1):57. 26093498 10.1186/s12978-015-0043-6 PMC4496821

[44] Boerma T , Ronsmans C , Melesse DY , et al. Global epidemiology of use of and disparities in caesarean sections. Lancet. 2018;392(10155):1341‐1348. 30322584 10.1016/S0140-6736(18)31928-7

[45] Salas Garcia MC , Yee AL , Gilbert JA , Dsouza M . Dysbiosis in children born by caesarean section. Ann Nutr Metab. 2018;73(Suppl 3):24‐32. 30041170 10.1159/000492168

[46] Huurre A , Kalliomaki M , Rautava S , Rinne M , Salminen S , Isolauri E . Mode of delivery – effects on gut microbiota and humoral immunity. Neonatology. 2008;93:236‐240. 18025796 10.1159/000111102

[47] Costello EK , Carlisle EM , Bik EM , Morowitz MJ , Relman DA . Microbiome assembly across multiple body sites in low‐birthweight infants. mBio. 2013;4:e00782‐e00813. 24169577 10.1128/mBio.00782-13 PMC3809564

[48] Chu DM , Ma J , Prince AL , Antony KM , Seferovic MD , Aagaard KM . Maturation of the infant microbiome community structure and function across multiple body sites and in relation to mode of delivery. Nat Med. 2017;23:314‐326. 28112736 10.1038/nm.4272 PMC5345907

[49] Drell T , Stsepetova J , Simm J , et al. The influence of different maternal microbial communities on the development of infant gut and oral microbiota. Sci Rep. 2017;7(1):9940. 28855595 10.1038/s41598-017-09278-y PMC5577157

[50] Mason S . Some aspects of gastric function in the newborn. Arch Dis Childhood. 1962;37(194):387‐391. 14470855 10.1136/adc.37.194.387 PMC2012883

[51] Jackson MA , Goodrich JK , Maxan ME , et al. Proton pump inhibitors alter the composition of the gut microbiota. Gut. 2016;65(5):749‐756. 26719299 10.1136/gutjnl-2015-310861 PMC4853574

[52] Shaffer M , Lozupone C . Prevalence and source of fecal and oral bacteria on infant, child, and adult hands. mSystems 2018;3(1):e00192‐17. 29359197 10.1128/mSystems.00192-17 PMC5768791

[53] Bosch A , de Steenhuijsen Piters WAA , van Houten MA , et al. Maturation of the infant respiratory microbiota, environmental drivers, and health consequences. A prospective cohort study. Am J Respir Crit Care Med. 2017;196(12):1582‐1590. 28665684 10.1164/rccm.201703-0554OC

[54] Baas‐Becking LGM . Geobiologie of Inleiding tot de Milieukunde. The Hague: Van Stokkun & Zoon; 1934.

[55] Xu X , He J , Xue J , et al. Oral cavity contains distinct niches with dynamic microbial communities. Environ Microbiol. 2015;17(3):699‐710. 24800728 10.1111/1462-2920.12502

[56] Ren W , Zhang Q , Liu X , et al. Exploring the oral microflora of preschool children. J Microbiol. 2017;55(7):531‐537. 28434085 10.1007/s12275-017-6474-8

[57] Mason MR , Chambers S , Dabdoub SM , Thikkurissy S , Kumar PS . Characterizing oral microbial communities across dentition states and colonization niches. Microbiome. 2018;6(1):67. 29631628 10.1186/s40168-018-0443-2 PMC5891995

[58] Marcotte H , Lavoie MC . Oral microbial ecology and the role of salivary immunoglobulin A. Microbiol Mol Biol Rev. 1998;62(1):71‐109. 9529888 10.1128/mmbr.62.1.71-109.1998 PMC98907

[59] Katharios‐Lanwermeyer S , Xi C , Jakubovics NS , Rickard AH . Mini‐review: Microbial coaggregation: ubiquity and implications for biofilm development. Biofouling. 2014;30:1235‐1251. 25421394 10.1080/08927014.2014.976206

[60] Hannig C , Hannig M , Kensche A , Carpenter G . The mucosal pellicle – an underestimated factor in oral physiology. Arch Oral Biol. 2017;80:144‐152. 28419912 10.1016/j.archoralbio.2017.04.001

[61] Whittaker CJ , Klier CM , Kolenbrander PE . Mechanisms of adhesion by oral bacteria. Annu Rev Microbiol. 1996;50:513‐552. 8905090 10.1146/annurev.micro.50.1.513

[62] Bunker JJ , Bendelac A . IgA responses to microbiota. Immunity. 2018;49(2):211‐224. 30134201 10.1016/j.immuni.2018.08.011 PMC6107312

[63] Kilian M , Reinholdt J , Lomholt H , Poulsen K , Frandsen EV . Biological significance of IgA1 proteases in bacterial colonization and pathogenesis: critical evaluation of experimental evidence. APMIS. 1996;104(1‐6):321‐338. 8703438 10.1111/j.1699-0463.1996.tb00724.x

[64] Kononen E , Jousimies‐Somer H , Bryk A , Kilp T , Kilian M . Establishment of streptococci in the upper respiratory tract: longitudinal changes in the mouth and nasopharynx up to 2 years of age. J Med Microbiol. 2002;51:723‐730. 12358062 10.1099/0022-1317-51-9-723

[65] Proctor DM , Fukuyama JA , Loomer PM , et al. A spatial gradient of bacterial diversity in the human oral cavity shaped by salivary flow. Nat Commun. 2018;9(1):681. 29445174 10.1038/s41467-018-02900-1 PMC5813034

[66] Gronroos L , Alaluusua S . Site‐specific oral colonization of mutans streptococci detected by arbitrarily primed PCR fingerprinting. Caries Res. 2000;34(6):474‐480. 11093021 10.1159/000016626

[67] Klein MI , Florio FM , Pereira AC , Hofling JF , Goncalves RB . Longitudinal study of transmission, diversity, and stability of *Streptococcus mutans* and *Streptococcus sobrinus* genotypes in Brazilian nursery children. J Clin Microbiol. 2004;42(10):4620‐4626. 15472319 10.1128/JCM.42.10.4620-4626.2004 PMC522380

[68] Kahharova D , Brandt BW , Buijs MJ , et al. Maturation of the oral microbiome in caries‐free toddlers: a longitudinal study. J Dent Res. 2020;99(2):159‐167. 31771395 10.1177/0022034519889015 PMC6977153

[69] Lindquist B , Emilson CG . Colonization of *Streptococcus mutans* and *Streptococcus sobrinus* genotypes and caries development in children to mothers harboring both species. Caries Res. 2004;38:95‐103. 14767165 10.1159/000075932

[70] Milgrom P , Riedy CA , Weinstein P , Tanner AC , Manibusan L , Bruss J . Dental caries and its relationship to bacterial infection, hypoplasia, diet, and oral hygiene in 6‐ to 36‐month‐old children. Community Dent Oral Epidemiol. 2000;28(4):295‐306. 10901409 10.1034/j.1600-0528.2000.280408.x

[71] Tanner ACR , Milgrom PM , Kent R Jr , et al. The microbiota of young children from tooth and tongue samples. J Dent Res. 2002;81(1):53‐57. 11824414 10.1177/002203450208100112

[72] Watanabe S , Dawes C . Salivary flow rates and salivary film thickness in five‐year‐old children. J Dent Res. 1990;69(5):1150‐1153. 2335647 10.1177/00220345900690050601

[73] Faust K , Sathirapongsasuti JF , Izard J , et al. Microbial co‐occurrence relationships in the human microbiome. PLoS Comput Biol. 2012;8(7):e1002606. 22807668 10.1371/journal.pcbi.1002606 PMC3395616

[74] Caufield PW , Cutter GR , Dasanayake AP . Initial acquisition of mutans streptococci by infants: evidence for a discrete window of infectivity. J Dent Res. 1993;72(1):37‐45. 8418105 10.1177/00220345930720010501

[75] van Loveren C , Buijs JF , ten Cate JM . Similarity of bacteriocin activity profiles of mutans streptococci within the family when the children acquire the strains after the age of 5. Caries Res. 2000;34(6):481‐485. 11093022 10.1159/000016627

[76] Lynch DJ , Villhauer AL , Warren JJ , et al. Genotypic characterization of initial acquisition of *Streptococcus mutans* in American Indian children. J Oral Microbiol. 2015;7:27182. 25840611 10.3402/jom.v7.27182 PMC4385128

[77] Pelto GH , Zhang Y , Habicht JP . Premastication: the second arm of infant and young child feeding for health and survival? Matern Child Nutr. 2010;6(1):4‐18. 20073131 10.1111/j.1740-8709.2009.00200.x PMC6860819

[78] Wan AK , Seow WK , Purdie DM , Bird PS , Walsh LJ , Tudehope DI . Oral colonization of *Streptococcus mutans* in six‐month‐old predentate infants. J Dent Res. 2001;80(12):2060‐2065. 11808762 10.1177/00220345010800120701

[79] Bockmann MR , Harris AV , Bennett CN , Odeh R , Hughes TE , Townsend GC . Timing of colonization of caries‐producing bacteria: an approach based on studying monozygotic twin pairs. Int J Dent. 2011;2011:571573. 22028714 10.1155/2011/571573 PMC3199088

[80] Kozai K , Nakayama R , Tedjosasongko U , et al. Intrafamilial distribution of mutans streptococci in Japanese families and possibility of father‐to‐child transmission. Microbiol Immunol. 1999;43(2):99‐106. 10229263 10.1111/j.1348-0421.1999.tb02380.x

[81] Liu Y , Zou J , Shang R , Zhou XD . Genotypic diversity of *Streptococcus mutans* in 3‐ to 4‐year‐old Chinese nursery children suggests horizontal transmission. Arch Oral Biol. 2007;52(9):876‐881. 17466259 10.1016/j.archoralbio.2007.03.004

[82] Alves AC , Nogueira RD , Stipp RN , et al. Prospective study of potential sources of *Streptococcus mutans* transmission in nursery school children. J Med Microbiol. 2009;58(4):476‐481. 19273644 10.1099/jmm.0.005777-0

[83] Lapirattanakul J , Nakano K . Mother‐to‐child transmission of mutans streptococci. Future Microbiol. 2014;9(6):807‐823. 25046526 10.2217/fmb.14.37

[84] da Silva BV , Freitas‐Fernandes LB , da Silva Fidalgo TK , et al. Mother‐to‐child transmission of *Streptococcus mutans*: a systematic review and meta‐analysis. J Dent. 2015;43:181‐191. 25486222 10.1016/j.jdent.2014.12.001

[85] Gronroos L , Saarela M , Matto J , Tanner‐Salo U , Vuorela A , Alaluusua S . Mutacin production by *Streptococcus mutans* may promote transmission of bacteria from mother to child. Infect Immun. 1998;66(6):2595‐2600. 9596721 10.1128/iai.66.6.2595-2600.1998 PMC108243

[86] Lin HK , Fang CE , Huang MS , et al. Effect of maternal use of chewing gums containing xylitol on transmission of mutans streptococci in children: a meta‐analysis of randomized controlled trials. Int J Paed Dent. 2016;26(1):35‐44. 10.1111/ipd.12155 25684114

[87] Tanner AC , Milgrom PM , Kent R Jr , et al. Similarity of the oral microbiota of pre‐school children with that of their caregivers in a population‐based study. Oral Microbiol Immunol. 2002;17(6):379‐387. 12485330 10.1034/j.1399-302x.2002.170608.x

[88] Kobayashi N , Ishihara K , Sugihara N , Kusumoto M , Yakushiji M , Okuda K . Colonization pattern of periodontal bacteria in Japanese children and their mothers. J Periodont Res. 2008;43(2):156‐161. 10.1111/j.1600-0765.2007.01005.x 18302616

[89] Tuite‐McDonnell M , Griffen AL , Moeschberger ML , Dalton RE , Fuerst PA , Leys EJ . Concordance of *Porphyromonas gingivalis* colonization in families. J Clin Microbiol. 1997;35(2):455‐461. 9003615 10.1128/jcm.35.2.455-461.1997 PMC229599

[90] Kononen E , Wolf J , Matto J , et al. The *Prevotella intermedia* group organisms in young children and their mothers as related to maternal periodontal status. J Periodont Res. 2000;35(6):329‐334. 10.1034/j.1600-0765.2000.035006329.x 11144405

[91] Kononen E , Kanervo A , Takala A , Asikainen S , Jousimies‐Somer H . Establishment of oral anaerobes during the first year of life. J Dent Res. 1999;78(10):1634‐1639. 10520968 10.1177/00220345990780100801

[92] Könönen E . Development of oral bacterial flora in young children. Ann Med. 2000;32(2):107‐112. 10766401 10.3109/07853890009011759

[93] Andreas NJ , Kampmann B , Mehring L‐D . Human breast milk: a review on its composition and bioactivity. Early Hum Dev. 2015;91(11):629‐635. 26375355 10.1016/j.earlhumdev.2015.08.013

[94] Verhasselt V . Is infant immunization by breastfeeding possible? Philos Trans R Soc Lond B Biol Sci. 2015;370(1671):20140139. 25964452 10.1098/rstb.2014.0139 PMC4527385

[95] Cacho NT , Lawrence RM . Innate immunity and breast milk. Front Immunol. 2017;8:584. 28611768 10.3389/fimmu.2017.00584 PMC5447027

[96] Witkowska‐Zimny M , Kaminska‐El‐Hassan E . Cells of human breast milk. Cell Mol Biol Lett. 2017;22:11. 28717367 10.1186/s11658-017-0042-4 PMC5508878

[97] Chow BD , Reardon JL , Perry EO , Laforce‐Nesbitt SS , Tucker R , Bliss JM . Host defense proteins in breast milk and neonatal yeast colonization. J Hum Lact. 2016;32:168‐173. 26116637 10.1177/0890334415592402 PMC5516210

[98] Moossavi S , Sepehri S , Robertson B , et al. Composition and variation of the human milk microbiota are influenced by maternal and early‐life factors. Cell Host Microbe. 2019;25(2):324‐335.e4. 30763539 10.1016/j.chom.2019.01.011

[99] Rodriguez JM . The origin of human milk bacteria: is there a bacterial entero‐mammary pathway during late pregnancy and lactation? Adv Nutr. 2014;5(6):779‐784. 25398740 10.3945/an.114.007229 PMC4224214

[100] Boix‐Amoros A , Collado MC , Mira A . Relationship between milk microbiota, bacterial load, macronutrients, and human cells during lactation. Front Microbiol. 2016;7:492. 27148183 10.3389/fmicb.2016.00492 PMC4837678

[101] Fitzstevens JL , Smith KC , Hagadorn JI , Caimano MJ , Matson AP , Brownell EA . Systematic review of the human milk microbiota. Nutr Clin Pract. 2017;32(3):354‐364. 27679525 10.1177/0884533616670150

[102] Al‐Shehri SS , Knox CL , Liley HG , et al. Breastmilk‐saliva interactions boost innate immunity by regulating the oral microbiome in early infancy. PLoS One. 2015;10(9):e0135047. 26325665 10.1371/journal.pone.0135047 PMC4556682

[103] WHO . Infant and young child feeding. Geneva, Switzerland: World Health Organization; 2018.

[104] Martin CR , Ling PR , Blackburn GL . Review of infant feeding: key features of breast milk and infant formula. Nutrients. 2016;8(5):279. 10.3390/nu8050279 PMC4882692 27187450

[105] Al‐Shehri SS , Sweeney EL , Cowley DM , et al. Deep sequencing of the 16S ribosomal RNA of the neonatal oral microbiome: a comparison of breast‐fed and formula‐fed infants. Sci Rep. 2016;6(1):38309. 27922070 10.1038/srep38309 PMC5138828

[106] Holgerson PL , Vestman NR , Claesson R , et al. Oral microbial profile discriminates breast‐fed from formula‐fed infants. J Ped Gastroenter Nutr. 2013;56(2):127‐136. 10.1097/MPG.0b013e31826f2bc6 PMC3548038 22955450

[107] Romani Vestman N , Timby N , Holgerson P , et al. Characterization and in vitro properties of oral lactobacilli in breastfed infants. BMC Microbiol. 2013;13(1):193. 23945215 10.1186/1471-2180-13-193 PMC3751747

[108] Sulyanto RM , Thompson ZA , Beall CJ , Leys EJ , Griffen AL . The predominant oral microbiota is acquired early in an organized pattern. Sci Rep. 2019;9(1):10550. 31332213 10.1038/s41598-019-46923-0 PMC6646312

[109] Marsh PD , Zaura E . Dental biofilm: ecological interactions in health and disease. J Clin Periodontol. 2017;44(Suppl 18):S12‐S22. 28266111 10.1111/jcpe.12679

[110] Gross EL , Beall CJ , Kutsch SR , Firestone ND , Leys EJ , Griffen AL . Beyond *Streptococcus mutans*: dental caries onset linked to multiple species by 16S rRNA community analysis. PLoS One. 2012;7(10):e47722. 23091642 10.1371/journal.pone.0047722 PMC3472979

[111] Fakhruddin KS , Ngo HC , Samaranayake LP . Cariogenic microbiome and microbiota of the early primary dentition: a contemporary overview. Oral Dis. 2019;25(4):982‐995. 29969843 10.1111/odi.12932

[112] Ollila P , Niemela M , Uhari M , Larmas M . Risk factors for colonization of salivary lactobacilli and Candida in children. Acta Odontol Scand. 1997;55(1):9‐13. 9083568 10.3109/00016359709091933

[113] Ersin NK , Eronat N , Cogulu D , Uzel A , Aksit S . Association of maternal‐child characteristics as a factor in early childhood caries and salivary bacterial counts. J Dent Child. 2006;73:105‐111. 16948372

[114] Amadio JVRDS , Hahn RC . Prevalence of *Candida* spp in the oral cavity of infants receiving artificial feeding and breastfeeding and the breasts of nursing mothers. J Pediatr Infect Dis. 2011;6:231‐236.

[115] Hannula J , Saarela M , Jousimies‐Somer H , et al. Age‐related acquisition of oral and nasopharyngeal yeast species and stability of colonization in young children. Oral Microbiol Immunol. 1999;14(3):176–182. 10495712 10.1034/j.1399-302x.1999.140306.x

[116] Hesselmar B , Sjoberg F , Saalman R , Aberg N , Adlerberth I , Wold AE . Pacifier cleaning practices and risk of allergy development. Pediatrics. 2013;131:e1829‐e1837. 23650304 10.1542/peds.2012-3345

[117] Mason MR , Preshaw PM , Nagaraja HN , Dabdoub SM , Rahman A , Kumar PS . The subgingival microbiome of clinically healthy current and never smokers. ISME J. 2015;9:268‐272. 25012901 10.1038/ismej.2014.114 PMC4274424

[118] Wu J , Peters BA , Dominianni C , et al. Cigarette smoking and the oral microbiome in a large study of American adults. ISME J. 2016;10:2435‐2446. 27015003 10.1038/ismej.2016.37 PMC5030690

[119] de Jong‐Lenters M , Duijster D , Schuller A , van Loveren C , Verrips E . Dental caries and externalizing behaviour problems in a high‐risk child population. Eur J Oral Sci. 2018;126:417‐425. 30051921 10.1111/eos.12542 PMC6175340

[120] Mashima I , Theodorea CF , Thaweboon B , Thaweboon S , Scannapieco FA , Nakazawa F . Exploring the salivary microbiome of children stratified by the oral hygiene index. PLoS One. 2017;12:e0185274. 28934367 10.1371/journal.pone.0185274 PMC5608389

[121] Volgenant CM , Zaura E , Brandt BW , et al. Red fluorescence of dental plaque in children – a cross‐sectional study. J Dent. 2017;58:40‐47. 28115186 10.1016/j.jdent.2017.01.007

[122] Goodrich JK , Davenport ER , Clark AG , Ley RE . The relationship between the human genome and microbiome comes into view. Annu Rev Genet. 2017;51:413‐433. 28934590 10.1146/annurev-genet-110711-155532 PMC5744868

[123] Goodman HO , Luke JE , Rosen S , Hackel E . Heritability in dental caries, certain oral microflora and salivary components. Am J Hum Genet. 1959;11:263‐273. 13851069 PMC1932006

[124] Corby PM , Bretz WA , Hart TC , Filho MM , Oliveira B , Vanyukov M . Mutans streptococci in preschool twins. Arch Oral Biol. 2005;50:347‐351. 15740714 10.1016/j.archoralbio.2004.08.010

[125] Corby PM , Bretz WA , Hart TC , et al. Heritability of oral microbial species in caries‐active and caries‐free twins. Twin Res Hum Genet. 2007;10:821‐828. 18179393 10.1375/twin.10.6.821 PMC3148892

[126] Hughes T , Bockmann M , Mihailidis S , et al. Genetic, epigenetic, and environmental influences on dentofacial structures and oral health: ongoing studies of Australian twins and their families. Twin Res Hum Genet. 2013;16:43‐51. 23394189 10.1017/thg.2012.78

[127] Ooi G , Townsend G , Seow WK . Bacterial colonization, enamel defects and dental caries in 4‐6‐year‐old mono‐ and dizygotic twins. Int J Paed Dent. 2014;24:152‐160. 10.1111/ipd.12041 23721206

[128] Gomez A , Espinoza JL , Harkins DM , et al. Host genetic control of the oral microbiome in health and disease. Cell Host Microbe 2017;22(3):269‐278.e3. 28910633 10.1016/j.chom.2017.08.013 PMC5733791

[129] Marsh PD . Microbial ecology of dental plaque and its significance in health and disease. Adv Dent Res. 1994;8(2):263‐271. 7865085 10.1177/08959374940080022001

[130] Demmitt BA , Corley RP , Huibregtse BM , et al. Genetic influences on the human oral microbiome. BMC Genom. 2017;18(1):659. 10.1186/s12864-017-4008-8 PMC5571580 28836939

[131] Stahringer SS , Clemente JC , Corley RP , et al. Nurture trumps nature in a longitudinal survey of salivary bacterial communities in twins from early adolescence to early adulthood. Genome Res. 2012;22:2146‐2152. 23064750 10.1101/gr.140608.112 PMC3483544

[132] Du Q , Li M , Zhou X , Tian K . A comprehensive profiling of supragingival bacterial composition in Chinese twin children and their mothers. Antonie Van Leeuwenhoek. 2017;110(5):615‐627. 28120199 10.1007/s10482-017-0828-4

[133] Blekhman R , Goodrich JK , Huang K , et al. Host genetic variation impacts microbiome composition across human body sites. Genome Biol. 2015;16(1):191. 26374288 10.1186/s13059-015-0759-1 PMC4570153

[134] Acton RT , Dasanayake AP , Harrison RA , et al. Associations of MHC genes with levels of caries‐inducing organisms and caries severity in African‐American women. Hum Immunol. 1999;60(10):984‐989. 10566600 10.1016/s0198-8859(99)00088-9

[135] Louvanto K , Roger M , Faucher MC , Syrjänen K , Grenman S , Syrjänen S . HLA‐G and vertical mother‐to‐child transmission of human papillomavirus infection. Hum Immunol. 2018;79(6):471‐476. 29544814 10.1016/j.humimm.2018.03.002

[136] Malamud D , Christensen CM , Navazesh M , Davis C . Bacterial agglutinin activity in the saliva of human identical and fraternal twins. Arch Oral Biol. 1988;33(11):801‐805. 3257085 10.1016/0003-9969(88)90104-5

[137] Rudney JD , Michalowicz BS , Krig MA , Kane PK , Pihlstrom BL . Genetic contributions to saliva protein concentrations in adult human twins. Arch Oral Biol. 1994;39(6):513‐517. 8067921 10.1016/0003-9969(94)90148-1

[138] Grotzinger AD , Mann FD , Patterson MW , et al. Twin models of environmental and genetic influences on pubertal development, salivary testosterone, and estradiol in adolescence. Clin Endocrinol. 2018;88(2):243‐250. 10.1111/cen.13522 PMC5771835 29161770

[139] Zaura E , Brandt BW , Prodan A , et al. On the ecosystemic network of saliva in healthy young adults. ISME J. 2017;11(5):1218‐1231. 28072421 10.1038/ismej.2016.199 PMC5475835

[140] Poole AC , Goodrich JK , Youngblut ND , et al. Human salivary amylase gene copy number impacts oral and gut microbiomes. Cell Host Microbe. 2019;25(4):553‐564.e7. 30974084 10.1016/j.chom.2019.03.001

[141] Barros SP , Offenbacher S . Epigenetics: connecting environment and genotype to phenotype and disease. J Dent Res. 2009;88(5):400‐408. 19493882 10.1177/0022034509335868 PMC3317936

[142] Lynch RJM . The primary and mixed dentition, post‐eruptive enamel maturation and dental caries: a review. Int Dent J. 2013;63:3‐13. 24283279 10.1111/idj.12074 PMC9375027

[143] Kimura S , Ooshima T , Takiguchi M , et al. Periodontopathic bacterial infection in childhood. J Periodontol. 2002;73(1):20‐26. 11846195 10.1902/jop.2002.73.1.20

[144] Umeda M , Miwa Z , Takeuchi Y , et al. The distribution of periodontopathic bacteria among Japanese children and their parents. J Periodont Res. 2004;39(6):398‐404. 10.1111/j.1600-0765.2004.00754.x 15491344

[145] Gizani S , Papaioannou W , Haffajee AD , Kavvadia K , Quirynen M , Papagiannoulis L . Distribution of selected cariogenic bacteria in five different intra‐oral habitats in young children. Int J Paediatr Dent. 2009;19(3):193‐200. 19207737 10.1111/j.1365-263X.2008.00956.x

[146] Papaioannou W , Gizani S , Haffajee AD , Quirynen M , Mamai‐Homata E , Papagiannoulis L . The microbiota on different oral surfaces in healthy children. Oral Microbiol Immunol. 2009;24(3):183‐189. 19416446 10.1111/j.1399-302X.2008.00493.x

[147] Rotimi VO , Salako NO , Divia M , Asfour L , Kononen E . Prevalence of periodontal bacteria in saliva of Kuwaiti children at different age groups. J Infect Public Health. 2010;3(2):76‐82. 20701895 10.1016/j.jiph.2010.02.002

[148] Crielaard W , Zaura E , Schuller AA , Huse SM , Montijn RC , Keijser BJF . Exploring the oral microbiota of children at various developmental stages of their dentition in the relation to their oral health. BMC Med Genomics. 2011;4(1):22. 21371338 10.1186/1755-8794-4-22 PMC3058002

[149] Brailsford SR , Sheehy EC , Gilbert SC , et al. The microflora of the erupting first permanent molar. Caries Res. 2005;39:78‐84. 15591739 10.1159/000081661

[150] Matsson L . Factors influencing the susceptibility to gingivitis during childhood – a review. Int J Paediatr Dent. 1993;3:119‐127. 8260459 10.1111/j.1365-263x.1993.tb00067.x

[151] Loomba‐Albrecht LA , Styne DM . The physiology of puberty and its disorders. Ped Ann. 2012;41:e1‐e9. 10.3928/00904481-20120307-08 22494212

[152] Kumarov P , Puberty AA . Physiology and Abnormalities. New York, NY: Springer; 2016.

[153] Kumar PS . Sex and the subgingival microbiome: do female sex steroids affect periodontal bacteria? Periodontol 2000. 2013;61(1):103‐124. 23240946 10.1111/j.1600-0757.2011.00398.x

[154] Romeo RD . Pubertal maturation and programming of hypothalamic‐pituitary‐adrenal reactivity. Front Neuroendocrinol. 2010;31(2):232‐240. 20193707 10.1016/j.yfrne.2010.02.004

[155] Dhabhar FS . The short‐term stress response – mother nature's mechanism for enhancing protection and performance under conditions of threat, challenge, and opportunity. Front Neuroendocrinol. 2018;49:175‐192. 29596867 10.1016/j.yfrne.2018.03.004 PMC5964013

[156] Swerdloff RS , Odell WD . Hormonal mechanisms in the onset of puberty. Postgrad Med J. 1975;51(594):200‐208. 1197148 10.1136/pgmj.51.594.200 PMC2495948

[157] Oskis A , Loveday C , Hucklebridge F , Thorn L , Clow A . Diurnal patterns of salivary cortisol across the adolescent period in healthy females. Psychoneuroendocrinology. 2009;34(3):307‐316. 18952383 10.1016/j.psyneuen.2008.09.009

[158] Mombelli A , Gusberti FA , van Oosten MA , Lang NP . Gingival health and gingivitis development during puberty. A 4‐year longitudinal study. J Clin Periodontol. 1989;16(7):451‐456. 2768539 10.1111/j.1600-051x.1989.tb01674.x

[159] Mombelli A , Lang NP , Burgin WB , Gusberti FA . Microbial changes associated with the development of puberty gingivitis. J Periodont Res. 1990;25:331‐338. 10.1111/j.1600-0765.1990.tb00924.x 2148945

[160] Gusberti FA , Mombelli A , Lang NP , Minder CE . Changes in subgingival microbiota during puberty. A 4‐year longitudinal study. J Clin Periodontol. 1990;17(10):685‐692. 2262580 10.1111/j.1600-051x.1990.tb01054.x

[161] Oh TJ , Eber R , Wang HL . Periodontal diseases in the child and adolescent. J Clin Periodontol. 2002;29(5):400‐410. 12060422 10.1034/j.1600-051x.2002.290504.x

[162] Wessels JM , Felker AM , Dupont HA , Kaushic C . The relationship between sex hormones, the vaginal microbiome and immunity in HIV‐1 susceptibility in women. Dis Model Mech. 2018;11(9):dmm035147. 30154116 10.1242/dmm.035147 PMC6177003

[163] Kaliannan K , Robertson RC , Murphy K , et al. Estrogen‐mediated gut microbiome alterations influence sexual dimorphism in metabolic syndrome in mice. Microbiome. 2018;6(1):205. 30424806 10.1186/s40168-018-0587-0 PMC6234624

[164] Kornman KS , Loesche WJ . Effects of estradiol and progesterone on *Bacteroides melaninogenicus* and *Bacteroides gingivalis* . Infect Immun. 1982;35(1):256‐263. 6119293 10.1128/iai.35.1.256-263.1982 PMC351023

[165] Yokoyama M , Hinode D , Masuda K , Yoshioka M , Grenier D . Effect of female sex hormones on *Campylobacter rectus* and human gingival fibroblasts. Oral Microbiol Immunol. 2005;20(4):239‐243. 15943769 10.1111/j.1399-302X.2005.00222.x

[166] Relloso M , Aragoneses‐Fenoll L , Lasarte S , et al. Estradiol impairs the Th17 immune response against *Candida albicans* . J Leukoc Biol. 2012;91:159‐165. 21965175 10.1189/jlb.1110645

[167] Jentsch HF , Marz D , Kruger M . The effects of stress hormones on growth of selected periodontitis related bacteria. Anaerobe. 2013;24:49‐54. 24036419 10.1016/j.anaerobe.2013.09.001

[168] Duran‐Pinedo AE , Solbiati J , Frias‐Lopez J . The effect of the stress hormone cortisol on the metatranscriptome of the oral microbiome. NPJ Biofilms Microbiomes. 2018;4:25. 30345066 10.1038/s41522-018-0068-z PMC6194028

[169] Kennedy B , Peura S , Hammar U , et al. Oral microbiota development in early childhood. Sci Rep. 2019;9(1):19025. 31836727 10.1038/s41598-019-54702-0 PMC6911045

[170] Johansson I , Witkowska E , Kaveh B , Lif Holgerson P , Tanner AC . The microbiome in populations with a low and high prevalence of caries. J Dent Res. 2016;95(1):80‐86. 26442950 10.1177/0022034515609554 PMC4700664

[171] Johansson I , Esberg A , Eriksson L , Haworth S , Lif HP . Self‐reported bovine milk intake is associated with oral microbiota composition. PLoS One. 2018;13(3):e0193504. 29561863 10.1371/journal.pone.0193504 PMC5862454

[172] Willis JR , Gonzalez‐Torres P , Pittis AA , et al. Citizen science charts two major "stomatotypes" in the oral microbiome of adolescents and reveals links with habits and drinking water composition. Microbiome. 2018;6(1):218. 30522523 10.1186/s40168-018-0592-3 PMC6284318

[173] Koopman JE , van der Kaaij NC , Buijs MJ , et al. The effect of fixed orthodontic appliances and fluoride mouthwash on the oral microbiome of adolescents – a randomized controlled clinical trial. PLoS One. 2015;10(9):e0137318. 26332408 10.1371/journal.pone.0137318 PMC4558009

[174] Lucchese A , Bondemark L , Marcolina M , Manuelli M . Changes in oral microbiota due to orthodontic appliances: a systematic review. J Oral Microbiol. 2018;10(1):1476645. 29988826 10.1080/20002297.2018.1476645 PMC6032020

[175] Papageorgiou SN , Xavier GM , Cobourne MT , Eliades T . Effect of orthodontic treatment on the subgingival microbiota: a systematic review and meta‐analysis. Orthod Craniofac Res. 2018;21(4):175‐185. 30028077 10.1111/ocr.12237

[176] Wilson M . The Human Microbiota in Health and Disease: An Ecological and Community‐Based Approach. Abingdon, UK: Taylor & Francis Ltd; 2018.

[177] Baker JL , Bor B , Agnello M , Shi W , He X . Ecology of the oral microbiome: beyond bacteria. Trends Microbiol. 2017;25(5):362‐374. 28089325 10.1016/j.tim.2016.12.012 PMC5687246

[178] Al‐Rusan RM , Darwazeh AM , Lataifeh IM . The relationship of *Candida* colonization of the oral and vaginal mucosae of mothers and oral mucosae of their newborns at birth. Oral Surg Pral Med Oral Pathol Oral Radiol. 2017;123(4):459‐463. 10.1016/j.oooo.2017.01.003 28283094

[179] Filippidi A , Galanakis E , Maraki S , et al. The effect of maternal flora on *Candida* colonisation in the neonate. Mycoses. 2014;57:43‐48. 23758480 10.1111/myc.12100

[180] Azevedo MJ , Pereira ML , Araujo R , Ramalho C , Zaura E , Sampaio‐Maia B . Influence of delivery and feeding mode in oral fungi colonization – a systematic review. Microb Cell. 2020;7(2):36‐45. 32025512 10.15698/mic2020.02.706 PMC6993125

[181] Stecksen‐Blicks C , Granstrom E , Silfverdal SA , West CE . Prevalence of oral *Candida* in the first year of life. Mycoses. 2015;58:550‐556. 26214300 10.1111/myc.12355

[182] Kadir T , Uygun B , Akyuz S . Prevalence of *Candida* species in Turkish children: relationship between dietary intake and carriage. Arch Oral Biol. 2005;50:33‐37. 15598415 10.1016/j.archoralbio.2004.07.004

[183] Matamala‐Valdes L , Sanchez‐Alonzo K , Parra C , Saez K , Aguayo‐Reyes A , Garcia A . Detection of intracellular *Helicobacter* *pylori* in Candida. SPP from neonate oral swabs. Rev Assoc Med Bras. 2018;64(10):928‐935. 30517241 10.1590/1806-9282.64.10.928

[184] Ward TL , Dominguez‐Bello MG , Heisel T , Al‐Ghalith G , Knights D , Gale CA . Development of the human mycobiome over the first month of life and across body sites. mSystems. 2018;3(3):e00140‐17. 10.1128/mSystems.00140-17 29546248 PMC5840654

[185] Fechney JM , Browne GV , Prabhu N , et al. Preliminary study of the oral mycobiome of children with and without dental caries. J Oral Microbiol. 2019;11(1):1536182. 30598729 10.1080/20002297.2018.1536182 PMC6225480

[186] Rizzetto L , De Filippo C , Cavalieri D . Richness and diversity of mammalian fungal communities shape innate and adaptive immunity in health and disease. Eur J Immunol. 2014;44(11):3166‐3181. 25257052 10.1002/eji.201344403

[187] Wylie KM , Weinstock GM , Storch GA . Emerging view of the human virome. Transl Res. 2012;160(4):283‐290. 22683423 10.1016/j.trsl.2012.03.006 PMC3701101

[188] Wylie KM , Mihindukulasuriya KA , Zhou Y , Sodergren E , Storch GA , Weinstock GM . Metagenomic analysis of double‐stranded DNA viruses in healthy adults. BMC Biol. 2014;12(1):71. 25212266 10.1186/s12915-014-0071-7 PMC4177058

[189] Robles‐Sikisaka R , Ly M , Boehm T , Naidu M , Salzman J , Pride DT . Association between living environment and human oral viral ecology. ISME J. 2013;7(9):1710. 23598790 10.1038/ismej.2013.63 PMC3749502

[190] Abeles SR , Robles‐Sikisaka R , Ly M , et al. Human oral viruses are personal, persistent and gender‐consistent. ISME J. 2014;8(9):1753–1767. 24646696 10.1038/ismej.2014.31 PMC4139723

[191] McCann A , Ryan FJ , Stockdale SR , et al. Viromes of one year old infants reveal the impact of birth mode on microbiome diversity. PeerJ. 2018;6:e4694. 29761040 10.7717/peerj.4694 PMC5944432

[192] Abeles SR , Pride DT . Molecular bases and role of viruses in the human microbiome. J Mol Biol. 2014;426(23):3892‐3906. 25020228 10.1016/j.jmb.2014.07.002 PMC7172398

[193] Edlund A , Santiago‐Rodriguez TM , Boehm TK , Pride DT . Bacteriophage and their potential roles in the human oral cavity. J Oral Microbiol. 2015;7(1):27423. 25861745 10.3402/jom.v7.27423 PMC4393417

[194] Wang J , Gao Y , Zhao F . Phage‐bacteria interaction network in human oral microbiome. Environ Microbiol. 2016;18(7):2143‐2158. 26036920 10.1111/1462-2920.12923

[195] Nguyen‐Hieu T , Khelaifia S , Aboudharam G , Drancourt M . Methanogenic archaea in subgingival sites: a review. APMIS. 2013;121(6):467‐477. 23078250 10.1111/apm.12015

[196] Chaudhary PP , Conway PL , Schlundt J . Methanogens in humans: potentially beneficial or harmful for health. Appl Microbiol Biotechnol. 2018;102(7):3095‐3104. 29497795 10.1007/s00253-018-8871-2

[197] Grine G , Boualam MA , Drancourt M *Methanobrevibacter smithii*, a methanogen consistently colonising the newborn stomach. Eur J Clin Microbiol Infect Dis. 2017;36(12):2449‐2455. 28823095 10.1007/s10096-017-3084-7

[198] Keppler F , Schiller A , Ehehalt R , Greule M , Hartmann J , Polag D . Stable isotope and high precision concentration measurements confirm that all humans produce and exhale methane. J Breath Res. 2016;10(1):16003. 10.1088/1752-7155/10/1/016003 26824393

[199] Bor B , Bedree JK , Shi W , McLean JS , He X . Saccharibacteria (TM7) in the human oral microbiome. J Dent Res. 2019;98(5):500‐509. 30894042 10.1177/0022034519831671 PMC6481004

[200] He X , McLean JS , Edlund A , et al. Cultivation of a human‐associated TM7 phylotype reveals a reduced genome and epibiotic parasitic lifestyle. Proc Natl Acad Sci USA. 2015;112(1):244. 25535390 10.1073/pnas.1419038112 PMC4291631

[201] Bonner M , Amard V , Bar‐Pinatel C , et al. Detection of the amoeba *Entamoeba gingivalis* in periodontal pockets. Parasite. 2014;21:30. 24983705 10.1051/parasite/2014029 PMC4077299

[202] Mielnik‐Blaszczak M , Rzymowska J , Michalowski A , Skawinska‐Bednarczyk A , Blaszczak J *Entamoeba gingivalis* – prevalence and correlation with dental caries in children from rural and urban regions of Lublin Province. Eastern Poland. Ann Agric Environ Med. 2018;25(4):656‐658. 30586973 10.26444/aaem/80403

[203] Wampach L , Heintz‐Buschart A , Hogan A , et al. Colonization and succession within the human gut microbiome by archaea, bacteria, and microeukaryotes during the first year of life. Front Microbiol. 2017;8:738. 28512451 10.3389/fmicb.2017.00738 PMC5411419

